# Development and validation of a person-centered antenatal care scale for low- and middle-income countries

**DOI:** 10.1038/s44294-026-00149-4

**Published:** 2026-06-08

**Authors:** Patience A. Afulani, Hawa Malechi, Daniel Enos Sekwo, Jaffer Okiring, Moro Ali, Osamuedeme J. Odiase, Beryl Ogolla, Joyceline Kinyua, Linnet Ongeri, Raymond A. Aborigo

**Affiliations:** 1 Department of Obstetrics, Gynecology, and Reproductive Sciences, University of California San Francisco, San Francisco, CA USA; 2 Department of Epidemiology & Biostatistics, University of California San Francisco, San Francisco, CA USA; 3 Department of Obstetrics and Gynecology, University for Development Studies, Tamale, Ghana; 4 Tamale Teaching Hospital, Tamale, Ghana; 5 Navrongo Health Research Center, Navrongo, Ghana; 6 Global Programs for Research and Training, Kampala, Uganda; 7 Department of Epidemiology and Biostatistics, School of Public Health, University of Technology and Applied Sciences, Navrongo, Ghana; 8 Global Programs for Research and Training, Nairobi, Kenya; 9 Kenya Medical Research Institute, Nairobi, Kenya

**Keywords:** Health care, Medical research

## Abstract

We aimed to develop and validate a tool to comprehensively measure person-centered antenatal care (PCANC) in low- and middle-income countries (LMICs). We followed standard procedures for scale development, including literature review, expert reviews, cognitive interviews, and pretesting to ensure content validity. Questions were iteratively revised at each stage and administered in surveys with pregnant and postpartum women in Ghana and Kenya. The survey data were used for psychometric analysis, resulting in a 36-item PCANC scale with three subscales: “dignity and respect,” “communication and autonomy,” and “responsive and supportive care.” The Cronbach’s alpha is 0.90 for the full scale and >0.7 for each subscale. The summative PCANC scores correlate with global measures of antenatal care quality, satisfaction, and future care location, suggesting good criterion validity. The PCANC scale has high validity and reliability and will facilitate efforts to measure and improve respectful and responsive antenatal care in LMICs.

## Introduction

High-quality antenatal care (ANC) is critical for preventing, identifying, and managing pregnancy complications to ensure the health of mothers and their babies^1^. The proportion of women receiving ANC has increased globally, including in low- and middle-income countries (LMICs), with over 80% of women in sub-Saharan Africa having at least one ANC contact during their pregnancy^2^. Although current World Health Organization (WHO) guidelines recommend eight antenatal contacts^1^, less than two-thirds of women in sub-Saharan Africa achieve the previously recommended four visits^2^. Additionally, significant quality gaps persist in both service provision and the ANC experience^3^, further contributing to the low coverage.

Person-centered care across the pregnancy, childbirth, and postpartum continuum is critical to maternal health from both a quality-of-care and human rights perspective^4,5^. We define person-centered antenatal care (PCANC) as ANC that is respectful, compassionate, and responsive to the needs, preferences, and values of pregnant women and gender-diverse people (women used subsequently for brevity)—adapted from the Institute of Medicine’s definition of patient-centered care^6^. PCANC has both direct and indirect effects on maternal and neonatal outcomes through its effect on patient care, engagement, and health-seeking behavior, including skilled attendance at birth^7,8^. The WHO recommendations for ANC highlight the importance of a positive experience^1^. Operationalizing these recommendations requires measuring women’s experiences to identify gaps and inform interventions. However, efforts to measure women’s ANC experiences have lagged, with most efforts focused on childbirth^9^.

Studies on ANC have primarily focused on frequency and timing^10–15^. Among the few that address quality of care, most focus on the content of ANC service provision, with relatively fewer on the person-centeredness of care^16–22^. This is due to the lack of validated measures for PCANC. A recent scoping review of measures aligned with the WHO recommendations for ANC found that existing measures prioritize clinical content of ANC, with measures addressing women’s ANC experiences notably lacking^23^. The few studies that include ANC experience measures, however, have highlighted gaps in PCANC. For example, a study in Kenya found that about one-third of women did not often understand the purposes of tests and medicines received and did not feel able to ask questions to the healthcare provider, only half reported being consistently asked if they had any questions, and about a third reported that they could never discuss issues in private^16^. While studies in LMICs have measured various aspects of PCANC, no validated PCANC scale exists for LMICs.

Similar to ANC, there were few validated quantitative measures of person-centered care during childbirth until recently^24–26^. However, increased interest in women’s childbirth experiences led to the development of several quantitative tools for this in the last decade^27^. Among the comprehensive validated tools for measuring women’s experiences during childbirth is the person-centered maternity care (PCMC) scale, initially developed and validated in Kenya and India^26,28–30^. The PCMC scale, which has domains for communication and autonomy, dignity and respect, and supportive care, has had widespread uptake, with subsequent validation and use in many countries in Africa, Asia, and America^31–36^. The scale has enabled us to extend research on the predictors and consequences of PCMC^37–39^, and has been used to evaluate interventions to improve PCMC^40,41^. Furthermore, the PCMC scale informed the development of person-centered family planning and abortion scales^42–44^, and notably, a person-centered prenatal (antenatal) care scale focusing on the experiences of people of color in the United States (the PCPC-US scale)^45^.

The PCPC-US scale, which has 34 items with three domains for communication and autonomy, dignity and respect, and responsive and supportive care, was developed in the United States (US) based on standard procedures for scale development but with a focus on the experiences of Black women and other people of color in the US. Psychometric analyses with samples of predominantly Black^45^ and Latina^46^ women in the US show that the scale has high validity and reliability. The PCPC-US, however, has not been validated in an LMIC. We seek to address this gap. Our aim in this study is to adapt the PCPC-US scale to make it relevant to LMIC contexts, with an initial validation in Ghana and Kenya. We refer to this scale for LMICs as a PCANC scale (as against PCPC) because the term ‘antenatal care’ is more commonly understood in LMICs than ‘prenatal care,’ although both refer to the same service during pregnancy.

## Results

The first set of results confirmed the tool’s *conceptual adequacy*. During the initial review of the items by the study team, we retained all 34 items from the PCPC-US scale on assessment of face validity—at face value, they all appeared relevant to the experiences of women everywhere. We also added 12 items we identified as relevant to ANC in LMICs from the literature, bringing the total number of items to 46. These 46 items were then sent out for expert reviews. During the expert review meeting, all items were deemed relevant and recommended to be maintained except for three questions. For the question, “Did providers knock on your room’s door and wait for a response before entering,” it was agreed by all that it was not relevant because in the setting, the provider is usually seated in the consulting room, and the patient walks in. This question was thus excluded at this stage. Several people also suggested removing the items on “*preferred clinic” and “preferred provider”* because they were generally not feasible in the setting and thus not considered highly relevant. However, a few recommended that they should be maintained. These two items were therefore marked for potential deletion later, depending on their performance in subsequent phases. All respondents rated the tool as comprehensive or very comprehensive, which, together with the individual item ratings, indicated high content validity. There were, however, various suggestions to reword some questions and to include questions on the physical and financial accessibility of services, as well as counseling on specific issues. This increased the number of items to over 60, although some questions recommended for inclusion were not framed in a way that could facilitate scoring as part of a scale (e.g., questions on how much it costs to pay for services).

During the cognitive interviews, most participants rated all items as important, with recommendations to improve the wording of some items and to reorder some questions to improve flow in the first round of interviews. No substantive changes were recommended during the second set of cognitive interviews. At the end of the cognitive interviews, there were 68 questions, including 47 questions related to the three conceptual domains from the PCPC-US scale: four questions on wait time for various services, six questions on communication regarding specific ANC services, seven questions on counseling on specific issues, and four questions on perceived physical and financial accessibility. These items were all included in the questionnaire for pretesting and administered in the survey.

The second set of results confirmed the tool’s *psychometric adequacy* based on the *initial validation sample*. The analytic sample for the initial psychometric analysis, using data from only Ghana, was 595 due to incomplete data from five respondents. The demographics of respondents in the analytic sample are shown in Table 1. The average age was 27 years, with 83% between 20 and 34 years old. Most respondents were married or partnered (93%) and had one to three children (73%). Most had less than a college education (86%) and earned or received less than 300 Ghana cedis (<$30 at the time) a month (76%). Distributions of other variables are shown in Table 1.

**Table 1:** Characteristics of the initial validation sample, Ghana, *N* = 595

*Characteristic*	*N (%)*
Age in years: Mean (SD)	26.94 (5.57)
Below 20 yrs	35 (5.9)
20–24	208 (35.0)
25–29	164 (27.6)
30–34	122 (20.5)
35–39	55 (9.2)
40 or more	11 (1.8)
Marital status	
Married/Partnered	553 (92.9)
Single	34 (5.7)
Widowed/divorced/separated	8 (1.3)
Parity	
None	75 (12.6)
One	195 (32.8)
Two	151 (25.4)
Three	90 (15.1)
Four	53 (8.9)
5 or more	31 (5.2)
Pregnant or given birth in the last 6 months	
Yes, currently pregnant	297 (49.9)
Yes, gave birth in the last 6 months	298 (50.1)
Months of pregnancy (pregnant participants)	
6 months	43 (14.5)
7 months	99 (33.4)
8 months	99 (33.4)
9 months	53 (17.9)
10 months	2 (0.7)
Months postpartum (postpartum participants)	
3 weeks or less	59 (19.8)
4–7 weeks	61 (20.5)
8–11 weeks	39 (13.1)
3 months	52 (17.4)
4 months	38 (12.8)
5 months	30 (10.1)
6 months	19 (6.4)
Highest grade completed at school	
None	29 (4.9)
Primary or less	128 (21.5)
Post-primary/vocational	196 (32.9)
Secondary	188 (31.6)
College/university	53 (8.9)
Refused to answer	1 (0.2)
Partner’s highest grade completed	
None	47 (7.9)
Primary or less	111 (18.7)
Post-primary/vocational	140 (23.5)
Secondary	151 (25.4)
College/University	111 (18.7)
Refused to answer	35 (5.9)
Occupation	
Farming	104 (17.5)
Trading/selling	107 (18.0)
Hairdressing/dressmaking/craftsmanship	142 (23.9)
Housewife/unemployed	144 (24.2)
Teacher/student	51 (8.6)
Other	47 (7.9)
Partner’s occupation	
Farming	189 (31.8)
Trading/selling	62 (10.4)
Hairdressing/dressmaking/craftsmanship	78 (13.1)
Unemployed	27 (4.5)
Teacher/student	76 (12.8)
Motor/driver/mechanic	65 (10.9)
Mason/electrician/plumbing	30 (5.0)
Other	68 (11.4)
Read and write	
No, cannot read and write	147 (24.7)
Yes, but with some difficulty	210 (35.3)
Yes, can read and write very well	238 (40.0)
Money earned/received in a month (Ghana cedis)	
None/undisclosed	64 (10.8)
100 or less	200 (33.6)
101–200	111 (18.7)
201–300	77 (12.9)
301–400	35 (5.9)
401–500	45 (7.6)
More than 500	63 (10.6)
Household wealth quintile	
First (poorest)	187 (31.4)
Second (poorer)	56 (9.4)
Third (middle)	129 (21.7)
Fourth (richer)	117 (19.7)
Fifth (richest)	106 (17.8)
Household member work in a health facility	
No	517 (87.0)
Yes	77 (13.0)
Religion	
Christian	535 (89.9)
Muslim	40 (6.7)
Traditionalist	18 (3.0)
Other	2 (0.3)
Ethnicity	
Nankani/Frafra	291 (48.9)
Kasem	211 (35.5)
Builsa	63 (10.6)
Others	30 (5.0)
Timing of first ANC	
2 months or less	224 (37.6)
3–4	273 (45.9)
5–6	83 (13.9)
7 or more	15 (2.5)
Reason for first ANC visit	
Just for a routine checkup	475 (79.8)
Because of a problem	120 (20.2)
Frequency of ANC visits	
2 or less	32 (5.4)
3–5	184 (30.9)
6–8	207 (34.8)
9 or more times	172 (28.9)
Highest-level ANC facility	
Government hospital	105 (17.6)
Government health center	195 (32.8)
Other lower-level facility	240 (40.3)
Private or mission facility	55 (9.2)
Highest-level provider received ANC from	
Doctor/medical assistant/physician assistant	28 (4.7)
Midwife/nurse	567 (95.3)

*N* Number (Frequency), *SD* Standard deviation, *ANC* Antenatal care.

Distributions for individual PCANC items for the initial sample are shown in Supplementary Table 1. In general, most questions had responses across the four response options. However, most of the negatively worded items (neglect, verbal abuse, discrimination, physical abuse, bribe) had very low frequencies of occurrence, with 94% or more responding “no never” to a negative occurrence. A few questions were also not applicable (NA) to all respondents, with > 30% NA for wait time to retrieve folder and family treated with respect, and >15% NA on wait time for drugs and labs, support when needed, support to deal with emotional wellbeing, and preferred ANC provider and clinic. The correlations between the full set of items varied widely, from negative to 0.8. Of note was that the wait time and negatively worded variables were negatively correlated with most other items.

The Kaiser-Meyer-Olkin (KMO) values ranged from 0.6 to 0.9, with an overall KMO of 0.85, indicating suitability for factor analysis. Initial exploratory factor analysis (EFA) with all 68 items yielded nine factors with eigenvalues greater than one, accounting for 81% of the cumulative variance, although the scree plot suggested three to four factors. Most items from the original PCPC-US scale loaded on the first two factors, and the items on financial and physical accessibility and counseling and communication for specific tests and procedures loaded on the other factors. Therefore, most of these items were removed at this point as the analysis confirmed they were conceptually different from the rest of the items. The six questions about specific examinations and tests were recoded to combine into two variables.

All the perceived wait time questions also loaded poorly with the rest of the items. However, because timeliness is important to responsiveness, we decided to retain the items on wait time to see the clinical provider, get labs done, and obtain medications from the pharmacy (because the team thought these were important sources of delay in the facility). We dropped the question on wait time for folders (following clarifications that while some facilities keep folders, in many cases, the antenatal record book that mothers keep is used; hence, mothers do not wait for their folders, explaining the high proportion of NA on that question). The two variables on wait time for labs and drugs were, however, recoded to one item on wait time to get labs or drugs because of the large proportion of NA responses for each. We also decided to combine the questions on whether they were asked about their emotional and mental wellbeing because they were interpreted similarly, had similar distributions, and were strongly correlated (*r* = 0.76). Other items from the initial PCPC-US scale that loaded poorly were retained at this stage, yielding 50 items for subsequent analysis.

EFA of the 50 items yielded five factors with eigenvalues greater than one, accounting for 78% of the cumulative variance. However, most items loaded on three factors, and the scree plot supported a single dominant factor. Most items had a loading of >0.3 on one of the three factors, and on the single factor when constrained to this factor structure (Supplementary Table 2). The negatively worded items (discrimination, physical abuse, bribery, and neglect), which had low frequencies in the sample, as noted above, continued to load poorly (<0.2) and had high uniqueness (>0.95), so they were dropped at this stage. In iterative EFAs, more items were dropped for various reasons to produce a shorter scale. This included removing items based on expert reviews for low relevance (preferred clinic and provider) and for loadings <0.3 and uniqueness >0.90 (pressured).

Further, for items that were strongly correlated or captured a similar concept, only one was retained, with the one that was conceptually broader or had more favorable loading and uniqueness maintained (e.g., for “explained medicines” and “understood medicines,” we kept “explained medicines;” for “explained examinations and procedures” and “understood examinations and procedures”; we kept “explained examinations and procedures”; for asked “if they had questions” and “encouraged to ask questions,” we kept “encouraged to ask questions”; for “could ask any questions” and “held back from asking questions,” we kept “could ask any question”; for “language they could understand” and “language level they could understand,” we kept “language level they could understand”; and for “showed they cared” and “best care” we kept “best care”). “Family respected” was also removed because of the high NA response rate (47%), as many respondents did not have family members with them. Some items with moderate loading ( < 0.3 but >0.2: privacy, verbal abuse, cleanliness, enough staff, time with the provider, asking about their birth plan, asking about physical wellbeing) were, however, retained in this phase to assess potential removal in subsequent phases. The wait time items were also retained despite poor loading because of the conceptual relevance of timeliness. This yielded 36 items.

**Fig. 1:**
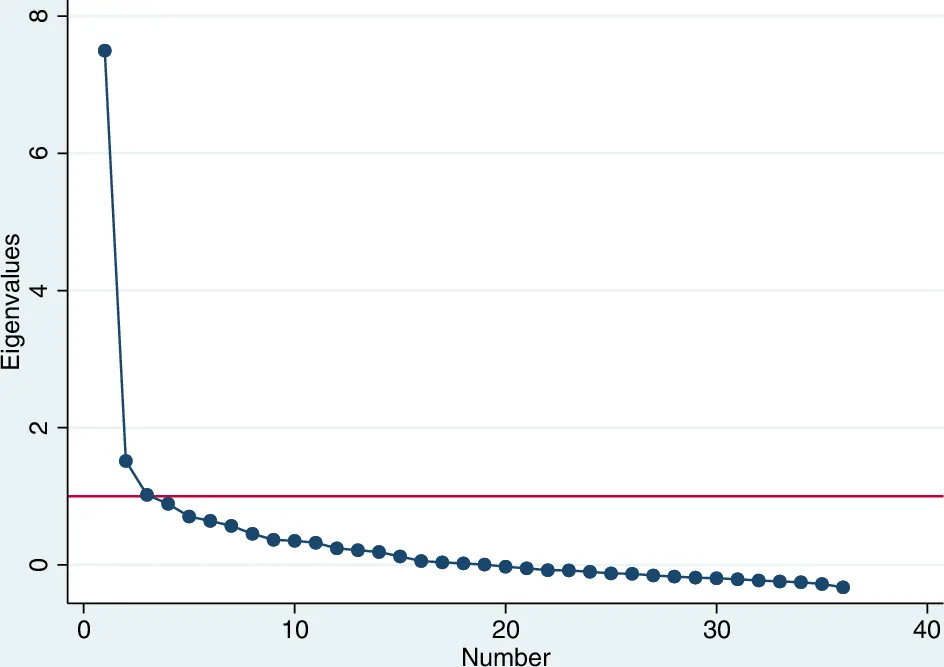
Scree plot from the initial validation sample. This scree plot is from exploratory factor analysis of the 36 person-centered antenatal care items with the initial validation sample.

EFA of the 36 items (Table 2) yielded three factors with eigenvalues greater than one, accounting for 81% of the cumulative variance, but with one dominant factor (Fig. 1) with an eigenvalue of 7.5, accounting for 61% of the cumulative variance. All items had loadings ≥0.3 on the three factors, except for the two wait time variables, which had loadings <0.2, and six items with loadings between 0.2 and 0.3 (privacy, verbal abuse, language level, time with provider, cleanliness, and enough staff). The uniqueness for most items was <0.9 except for five of these items, with a uniqueness of 0.95 for the wait time variables in the three-factor structure (Table 2). When the analysis was restricted to the single factor structure, all items had loadings of >0.3 except 10 items (the two wait time variables had loadings less than 0.2; and eight items—privacy, verbal abuse, time with provider, cleanliness, enough, birth plan, told exams, told results—had loadings between 0.2 and 0.3). The best fit in this sample was thus 28 items for a multidimensional scale and 26 for a unidimensional scale. Sequential exclusion of all 10 items with low loading in the three or single-factor structure did not significantly change the Cronbach’s alpha, and all versions had correlations >0.96. Thus, we proceeded with the 36 items for testing in subsequent samples.

**Table 2:** Exploratory factor analysis of retained 36 PCANC items with the initial validation sample, Ghana, *N* = 595

	Three-factor structure (full scale)				Single-factor structure (full scale)		Single-factor (subscales)	
*Subscale/items*	*Factor1*	*Factor2*	*Factor3*	*Uniqueness*	*Factor1*	*Uniqueness*	*Factor1*	*Uniqueness*
**Dignity and respect**								
Greeted	0.38		0.39	0.60	0.56	0.69	0.52	0.73
Treat you with respect	0.73			0.50	0.65	0.58	0.76	0.43
Friendly care	0.70			0.50	0.67	0.56	0.78	0.39
Knowledge valued	0.71			0.52	0.65	0.58	0.64	0.59
Privacy/not exposed			0.27	0.90	0.23	0.95	0.24	0.94
Info confidentiality			0.53	0.66	0.42	0.82	0.41	0.83
Auditory privacy	0.47			0.70	0.49	0.76	0.50	0.75
Verbal abuse	0.27			0.91	0.30	0.91	0.29	0.92
**Communication and autonomy**								
Introductions by provider			0.35	0.72	0.47	0.78	0.48	0.77
Called preferred name			0.32	0.83	0.34	0.88	0.33	0.89
Heard and listened to	0.66			0.60	0.58	0.67	0.49	0.76
Involved in decisions			0.38	0.69	0.49	0.76	0.47	0.78
Consent	0.63			0.64	0.55	0.70	0.53	0.72
Explain exams/procedures	0.49			0.68	0.53	0.72	0.57	0.68
Explain medications	0.40			0.75	0.49	0.76	0.55	0.69
Told examination results		0.34	0.40	0.75	0.19	0.96	0.26	0.93
Told test results		0.35	0.39	0.75	0.16	0.97	0.22	0.95
Could ask any questions	0.42			0.76	0.46	0.79	0.46	0.78
Encouraged questions	0.34	0.45		0.59	0.55	0.69	0.61	0.63
Questions were answered	0.33	0.33		0.65	0.56	0.68	0.59	0.66
Check understood	0.34	0.30		0.60	0.61	0.63	0.64	0.60
Language level they understood			0.24	0.85	0.34	0.88	0.32	0.89
Asked about birth plan		0.32		0.88	0.24	0.94	0.27	0.93
**Responsive and supportive care**								
Wait time for provider			0.11	0.98	0.10	0.99	0.12	0.99
Wait time for labs and drugs			0.12	0.95	-0.12	0.99	-0.14	0.98
Time with provider	0.28			0.92	0.20	0.96	0.17	0.97
Received best care	0.58			0.58	0.63	0.61	0.63	0.60
Physical wellbeing assessed	0.30			0.75	0.47	0.77	0.51	0.74
Mental/emotional wellbeing assessed		0.67		0.57	0.38	0.85	0.46	0.79
Resources for emotional/mental wellbeing		0.60		0.61	0.42	0.83	0.54	0.71
Attention when needed help		0.31		0.79	0.42	0.82	0.44	0.80
Trust	0.50			0.68	0.56	0.69	0.58	0.67
Competence	0.57			0.56	0.65	0.58	0.63	0.60
Cleanliness			0.25	0.87	0.19	0.96	0.22	0.95
Safe	0.40			0.77	0.38	0.86	0.40	0.84
Enough staff		0.28		0.88	0.22	0.95	0.29	0.92

*PCANC* Person-centered antenatal care.

As in prior validation studies, because the loading of the items does not group into clean conceptual categories, we created subscales based on conceptual groupings adapted from the WHO’s experience of care domains^47^. This gives three subscales for “dignity and respect,” “communication and autonomy,” and “responsive and supportive care.” EFA of items in each subscale yielded a single factor, with all items loading at >0.4 on the single factor and with uniqueness <0.8, except for the 10 items noted above (Table 2).

The full 36-item scale has a Cronbach’s alpha of 0.89, with an average interitem correlation of 0.18. The Cronbach’s alphas for the subscales are 0.74, 0.79, and 0.70 for the “dignity and respect,” “communication and autonomy,” and “responsive and supportive care” subscales, respectively, with average interitem correlations of 0.27, 0.20, and 0.15, respectively (Supplementary Table 3). When the wait time and time with provider variables are excluded, the Cronbach’s alpha does not change, but the average interitem correlations increase to 0.20 and 0.22 for the full scale and the “responsive and supportive care” subscale, respectively (i.e., 33-item scale).

The average standardized score for the 36-item PCANC scale is 77.6 out of 100, with scores of 86.4, 73.5, and 76.8. respectively for the “dignity and respect,” “communication and autonomy,” and “responsive and supportive care” subscales for the Ghana test sample (Supplementary Table 3). Cross-tabulating and regressing the full scale on patients’ ratings of satisfaction with ANC services, general perceived quality of ANC, and whether they would receive ANC in the same facility if they were to be pregnant again showed increasing PCANC scores with higher ratings of satisfaction, perceived quality of care, and intent to receive ANC in the same facility in the future (Table 3).

**Table 3:** Crosstabulation and linear regression on the PCANC score with the initial validation sample, Ghana, *N* = 595

	*Crosstab*			*Linear Regression*			
	*N*	Mean	SD	Coefficient	*p* value	[95% CI]	
**Satisfaction with ANC**							
Dissatisfied	5	54.44	21.4	−19.28	0.03	−36.17	−2.38
Neither satisfied nor dissatisfied	19	57.26	13.1	−16.46	0.00	−22.36	−10.57
Satisfied	284	73.72	11.0	ref.			
Very satisfied	287	83.12	8.54	9.40	0.00	7.78	11.03
**Perceived quality of ANC**							
Very poor	2	43.06	29.5	−27.34	0.07	−56.46	1.79
Poor	9	54.12	13.4	−16.28	0.00	−24.73	−7.82
Fair	20	59.49	13.4	−10.90	0.00	−16.85	−4.95
Good	183	70.39	10.5	ref.			
Very good	236	80.61	8.34	10.22	0.00	8.36	12.09
Excellent	143	86.23	5.93	15.84	0.00	14.03	17.66
**Will return for ANC in future**							
No, never	63	70.56	15.3	-9.07	0.00	−12.96	−5.18
Yes, somewhat	56	67.92	12.5	−11.71	0.00	−15.09	−8.33
Yes, definitely	475	79.63	10.3	ref			

*PCANC* Person-centered antenatal care, *ANC* Antenatal care, *N* Number (Frequency), *SD* Standard deviation, *95% CI* 95% confidence interval.

The third set of results confirmed the tool’s *psychometric adequacy* based on the *confirmation sample*. Among women participating in the “Caring for Providers to Improve Patient Experience” (CPIPE) trial baseline survey, 1992 received ANC, but four respondents had incomplete PCANC data, yielding an analytic sample of 1988 for the confirmation sample. The demographics of this sample, by country, are shown in Table 4. The average age was 26.9 (SD = 5.8) and 25.4 (SD = 5.6) years for Ghana and Kenya, respectively. Most respondents were married or partnered (91.5% and 79.8% for Ghana and Kenya, respectively) and had one to two children (55.4% and 51.7% for Ghana and Kenya, respectively). Only about 10% of respondents had a college education (9.5% and 11% for Ghana and Kenya, respectively), and most earned or received <$100 a month (61.3% with 1000 or less Ghana cedis for Ghana and 68.7% with 10,000 or less Kenyan Shillings (KSH) for Kenya).

**Table 4:** Characteristics of the confirmation sample, Ghana and Kenya, *N* = 1988

Characteristic	Overall (*N* = 1988)	Country		
		Kenya (*N* = 996)	Ghana (*N* = 992)	*p*
	*N* (%)	*N* (%)	*N* (%)	
Age in years: Mean (SD)	26.20 (5.78)	25.45 (5.65)	26.96 (5.80)	<0.001
Below 20 years	216 (10.9)	133 (13.4)	83 (8.4)	<0.001
20–24	683 (34.4)	384 (38.6)	299 (30.1)	
25–29	538 (27.1)	243 (24.4)	295 (29.7)	
30–34	338 (17.0)	148 (14.9)	190 (19.2)	
35–39	165 (8.3)	72 (7.2)	93 (9.4)	
40 or more	38 (1.9)	12 (1.2)	26 (2.6)	
Refused to answer	10 (0.5)	4 (0.4)	6 (0.6)	
Marital status				
Single	259 (13.0)	177 (17.8)	82 (8.3)	<0.001
Married/partnered	1702 (85.6)	795 (79.8)	907 (91.4)	
Widowed/divorced/separated	25 (1.3)	23 (2.3)	2 (0.2)	
Refused to answer	2 (0.1)	1 (0.1)	1 (0.1)	
Parity				
1–2	1063 (53.5)	515 (51.7)	548 (55.2)	0.006
3–4	652 (32.8)	322 (32.3)	330 (33.3)	
5–6	214 (10.8)	118 (11.8)	96 (9.7)	
7 or more	59 (3.0)	41 (4.1)	18 (1.8)	
Months of postpartum				
3 weeks or less	875 (44.0)	410 (41.2)	465 (46.9)	<0.001
4–7 weeks	560 (28.2)	310 (31.2)	250 (25.2)	
8–11 weeks	447 (22.5)	236 (23.7)	211 (21.3)	
3 months	105 (5.3)	39 (3.9)	66 (6.7)	
Highest grade completed at school				
None	166 (8.4)	3 (0.3)	163 (16.4)	<0.001
Primary or less	643 (32.3)	476 (47.8)	167 (16.8)	
Post-primary/vocational	255 (12.8)	53 (5.3)	202 (20.4)	
Secondary	719 (36.2)	354 (35.5)	365 (36.8)	
College/university	205 (10.3)	110 (11.0)	95 (9.6)	
Partner’s highest grade completed				
None	138 (6.9)	3 (0.3)	135 (13.6)	<0.001
Primary or less	504 (25.4)	301 (30.2)	203 (20.5)	
Post-primary/vocational	195 (9.8)	103 (10.3)	92 (9.3)	
Secondary	486 (24.4)	212 (21.3)	274 (27.6)	
College/university	330 (16.6)	142 (14.3)	188 (19.0)	
Refused to answer	285 (14.3)	199 (20.0)	86 (8.7)	
Don’t Know	50 (2.5)	36 (3.6)	14 (1.4)	
Occupation				
Farming	256 (12.9)	127 (12.8)	129 (13.0)	<0.001
Trading/selling	352 (17.7)	190 (19.1)	162 (16.3)	
Hairdressing/dressmaking/craftsmanship	233 (11.7)	50 (5.0)	183 (18.4)	
Housewife/unemployed	725 (36.5)	464 (46.6)	261 (26.3)	
Teacher/student	241 (12.1)	128 (12.9)	113 (11.4)	
Others	181 (9.1)	37 (3.7)	144 (14.5)	
Partner’s occupation				
Farming	538 (27.1)	194 (19.5)	344 (34.7)	<0.001
Trading/selling	249 (12.5)	156 (15.7)	93 (9.4)	
Hairdressing/dressmaking/craftsmanship	229 (11.5)	145 (14.6)	84 (8.5)	
unemployed	108 (5.4)	51 (5.1)	57 (5.7)	
Teacher/student	178 (9.0)	64 (6.4)	114 (11.5)	
Motor/driver/mechanic	335 (16.9)	229 (23.0)	106 (10.7)	
Mason/electrician/plumbing	10 (0.5)	4 (0.4)	6 (0.6)	
Others	341 (17.2)	153 (15.4)	188 (19.0)	
Read and write				
No, cannot read and write	322 (16.2)	16 (1.6)	306 (30.8)	<0.001
Yes, but with some difficulty	310 (15.6)	128 (12.9)	182 (18.3)	
Yes, can read and write very well	1350 (67.9)	849 (85.2)	501 (50.5)	
Refused to answer	6 (0.3)	3 (0.3)	3 (0.3)	
Money earned/received in a month (Ghana)				
≤1000 cedis		-	605 (61.2)	.
>1000 to 5000 cedis		-	153 (15.5)	
>5000 to 10,000 cedis		-	6 (0.6)	
Refused to answer		-	224 (22.7)	
Money earned/received in a month (Kenya)				
≤10,000 KSH		684 (68.7)	-	.
>10,000 to 50,000 KSH		229 (23.0)	-	
>50,000 to 100,000 KSH		12 (1.2)	-	
>100,000 to 150,000 KSH		2 (0.2)	-	
>200,000 KSH		1 (0.1)	-	
Refused to answer		67 (6.7)	-	
Household wealth quintile				
First	405 (20.4)	153 (15.4)	252 (25.4)	<0.001
Second	510 (25.7)	273 (27.4)	237 (23.9)	
Third	307 (15.4)	175 (17.6)	132 (13.3)	
Fourth	438 (22.0)	216 (21.7)	222 (22.4)	
Fifth	328 (16.5)	179 (18.0)	149 (15.0)	
Religion				
Christian	1698 (85.4)	980 (98.4)	718 (72.4)	<0.001
Muslim	264 (13.3)	11 (1.1)	253 (25.5)	
Traditionalist	24 (1.2)	5 (0.5)	19 (1.9)	
Other	2 (0.1)	0 (0.0)	2 (0.2)	
Ethnicity in Kenya				
Luyya		30 (3.0)	-	.
Kuria		95 (9.5)	-	
Abusuba		5 (0.5)	-	
Kisii		29 (2.9)	-	
Luo		815 (81.8)	-	
Other		22 (2.2)	-	
Ethnicity in Ghana				
Kasem		-	69 (7.0)	.
Nankani/Frafra		-	309 (31.1)	
Builsa		-	149 (15.0)	
Nabdam		-	4 (0.4)	
Kusal		-	82 (8.3)	
Talensi		-	50 (5.0)	
Mampruli		-	150 (15.1)	
Other		-	178 (17.9)	
Refused to answer		-	1 (0.1)	
Timing of first ANC				
2 months or less	526 (26.5)	116 (11.7)	410 (41.3)	<0.001
3–4	854 (43.0)	432 (43.4)	422 (42.5)	
5–6	519 (26.1)	394 (39.6)	125 (12.6)	
7 or more	88 (4.4)	53 (5.3)	35 (3.5)	
Reason for first ANC visit				
Because of a problem	301 (15.1)	187 (18.8)	114 (11.5)	<0.001
Just for a routine checkup	1681 (84.6)	803 (80.7)	878 (88.5)	
Don’t know	4 (0.2)	4 (0.4)	0 (0.0)	
Not applicable	1 (0.1)	1 (0.1)	0 (0.0)	
Number of ANC visits				
2 or less	91 (4.6)	72 (7.2)	19 (1.9)	<0.001
3–5	859 (43.2)	683 (68.6)	176 (17.7)	
6–8	637 (32.0)	226 (22.7)	411 (41.4)	
9 or more times	387 (19.5)	10 (1.0)	377 (38.0)	
Don’t know/missing	14 (0.7)	5 (0.5)	9 (0.9)	
Highest-level ANC facility				
Gov’t hospital	851 (43.0)	572 (57.5)	279 (28.4)	<0.001
Gov’t health center/other lower level	961 (48.6)	398 (40.0)	563 (57.3)	
Private or mission facility	165 (8.3)	24 (2.4)	141 (14.3)	
Highest-level provider received ANC from				
Doctor/medical assistant/clinical officer	343 (17.3)	256 (25.7)	87 (8.8)	<0.001
Nurse/midwife	1639 (82.6)	738 (74.2)	901 (91.0)	
Non-skilled attendant	3 (0.2)	1 (0.1)	2 (0.2)	

*N* Number (Frequency), *SD* Standard deviation. *ANC* Antenatal care. *KSH* Kenyan Shillings

Forty-six PCANC questions were included in the CPIPE trial baseline surveys, including the 36 items from the psychometric analysis, nine other items that the team thought were important to reassess in a new sample, and a new question on the accessibility of washrooms because some respondents noted there was no washroom in the facility in response to the question on cleanliness in the previous survey. The distribution of these items in this sample is shown in Supplementary Table 4. An initial EFA with this sample confirmed that the nine items that had performed poorly in the initial Ghana sample continued to perform poorly in the validation sample (Supplementary Table 5), so they were finally dropped. In addition, the item on time with a provider continued to perform poorly in this sample, with a uniqueness of one on the single-factor structure. So, this was also dropped at this point. The two wait time questions, however, loaded adequately with the three-factor structure, although they were the only two items loading on the third factor and loaded poorly in the single-factor structure. The new question about washroom accessibility also performed well. A decision was thus made to retain these three items, which maintained a 36-item scale.

**Fig. 2:**
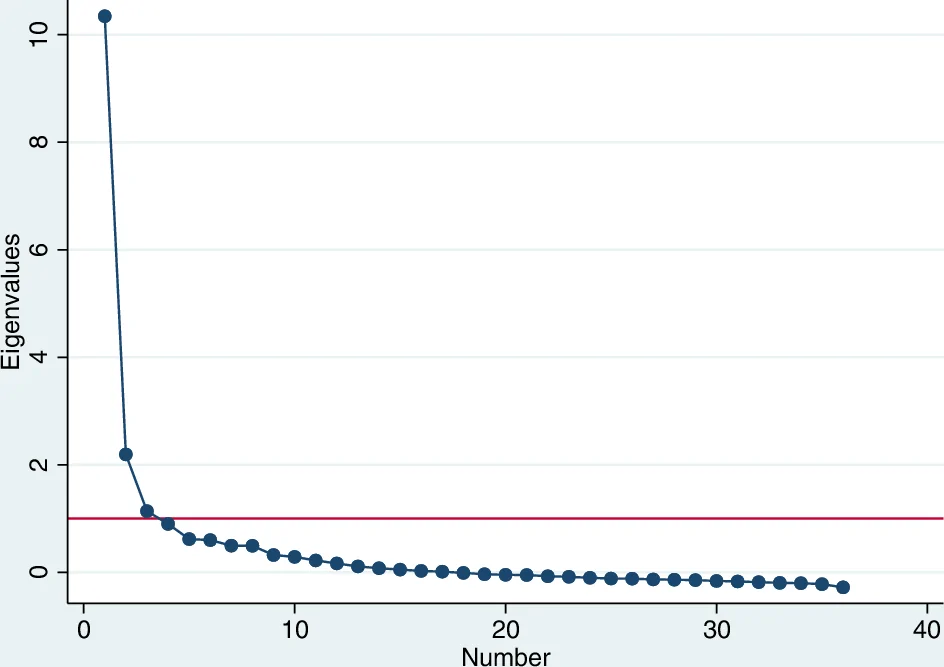
Scree plot from the confirmation sample. This scree plot is from exploratory factor analysis of the 36 person-centered antenatal care items with the confirmation sample.

The average KMO for the 36 items is 0.94 (range of 0.56 to 0.97), with three factors with eigenvalues greater than one accounting for 87% of the cumulative variance, and one dominant factor accounting for 66% of the cumulative variance (Fig. 2). All 36 remaining items loaded at >0.3 on the three factors, except for verbal abuse and auditory privacy, which had loadings between two and three (Supplementary Table 6), but were retained because of their strong conceptual relevance. As in the initial analysis, we created the subscales based on conceptual groupings for “dignity and respect,” “communication and autonomy,” and “responsive and supportive care.” EFA of items in each subscale yielded a single factor, with all items loading at >0.4 on the single factor and with uniqueness <0.8, except for verbal abuse, language, and the time variables (Supplementary Table 6). Given the poor loading of the two wait time variables, we also tested a 34-item scale that excludes them. Further, to provide a smaller subset of items for instances where all 36 items might not be feasible to administer, we used an iterative approach where all items with factor loading <0.3 were dropped, and additional items dropped based on our assessment of the strength of their relevance and similarity to other retained items, leading to a 20-item short scale (items shown in Table 5).

**Table 5:** Unstandardized and standardized loadings from the three-factor confirmatory model of the PCANC scale, Ghana and Kenya, *N* = 1988

Latent variable / observed variable	36 items		20 items	
	Unstandardized (SE)	Standardized (SE)	Unstandardized (SE)	Standardized (SE)
**Dignity and respect**				
*Greeted*	1	0.62 (0.02)	1	0.64 (0.02)
*Treat you with respect*	0.77 (0.03)	0.69 (0.02)	0.77 (0.03)	0.72 (0.02)
*Friendly care*	0.82 (0.03)	0.70 (0.02)	0.82 (0.03)	0.72 (0.02)
Knowledge valued	1.09 (0.05)	0.75 (0.02)	-	-
*Privacy not exposed*	0.91 (0.05)	0.53 (0.02)	0.87 (0.05)	0.53 (0.02)
*Info confidentiality*	0.59 (0.04)	0.47 (0.02)	0.58 (0.03)	0.48 (0.02)
Auditory privacy	0.85 (0.05)	0.45 (0.02)	-	-
Verbal abuse	0.16 (0.02)	0.23 (0.02)	-	-
**Communication and autonomy**				
*Introductions by provider*	1	0.34 (0.02)	1	0.34 (0.02)
*Called preferred name*	0.99 (0.10)	0.34 (0.02)	1.00 (0.10)	0.35 (0.02)
*Heard and listened to*	1.57 (0.12)	0.62 (0.02)	1.59 (0.12)	0.63 (0.02)
*Consent*	1.66 (0.12)	0.53 (0.02)	1.68 (0.12)	0.54 (0.02)
*Involved in decisions*	2.03 (0.15)	0.68 (0.01)	2.10 (0.15)	0.71 (0.02)
*Explain exams/procedures*	2.24 (0.16)	0.69 (0.02)	1.96 (0.15)	0.62 (0.02)
*Explain medications*	2.14 (0.15)	0.71 (0.01)	2.00 (0.14)	0.67 (0.02)
Told examination results	1.86 (0.14)	0.57 (0.02)	-	-
Told test results	1.94 (0.15)	0.60 (0.02)	-	-
Could ask any questions	1.93 (0.14)	0.68 (0.01)	-	-
*Encouraged questions*	2.36 (0.17)	0.72 (0.01)	2.17 (0.16)	0.66 (0.02)
Questions were answered	1.78 (0.13)	0.68 (0.02)	-	-
*Check understood information*	2.27 (0.16)	0.72 (0.01)	2.23 (0.16)	0.71 (0.01)
language they understood	0.35 (0.04)	0.25 (0.02)	-	-
Asked about birth plan	1.37 (0.11)	0.40 (0.02)	-	-
**Responsive and supportive care**				
*Received best care*	1	0.76 (0.01)	1	0.65 (0.02)
Wait time for provider	0.12 (0.03)	0.08 (0.02)	-	-
Wait time for labs or drugs	-0.18 (0.04)	-0.10 (0.02)	-	-
Physical wellbeing assessed	0.82 (0.05)	0.43 (0.02)	-	-
*Mental/emotional wellbeing assessed*	1.28 (0.06)	0.55 (0.02)	1.21 (0.07)	0.44 (0.02)
Resources for emotional/mental wellbeing	1.02 (0.06)	0.45 (0.02)	-	-
Attention when needed help	0.98 (0.04)	0.69 (0.01)	-	-
*Trust*	1.01 (0.03)	0.71 (0.01)	0.98 (0.03)	0.59 (0.02)
Competence	0.73 (0.03)	0.57 (0.02)	-	-
*Washroom availability*	0.58 (0.05)	0.31 (0.02)	0.52 (0.05)	0.24 (0.02)
*Cleanliness*	0.78 (0.05)	0.44 (0.02)	0.75 (0.05)	0.36 (0.02)
*Safe*	0.88 (0.03)	0.64 (0.02)	0.74 (0.03)	0.46 (0.02)
Enough staff	0.72 (0.04)	0.39 (0.02)	-	-

*PCANC* Person-centered antenatal care, *SE* Standard error.

Italicized items are those retained in 20-item short PCANC scale.

The 36-item and 20-item scales, with the three subscales, were then evaluated in Confirmatory Factor Analysis (CFA) for the combined sample, and for each country. The standardized factor loadings from the CFA for the observed 36 items mainly ranged from 0.08 to 0.76, with only one item (wait time for labs or drugs) having a negative factor loading, and almost all loadings >0.3, except for four items (verbal abuse, language they understood, and wait time for provider and labs or drugs) (Table 5). The model for Ghana had factor loadings ranging from −0.84 to 0.84, and for Kenya, from 0.08 to 0.71 for the 36 items (Supplementary Table 7). All items had standardized loadings >0.3 for the 20 items except for one item (washroom availability; Table 5). The Goodness-of-Fit indicators of the three-factor confirmatory model indicated that the model with the covariances of the error terms of 10 or more with the combined sample was the best-fitting model, as indicated by a root mean square error of approximation (RMSEA) of 0.048, *p* of close fit (*p* close) of 0.922, comparative fit index (CFI) of 0.941, Tucker-Lewis index (TLI) of 0.903, and Standardized root mean squared residual (SRMR) of 0.046 (Table 6 and Supplementary Table 8).

**Table 6:** Goodness-of-fit indicators for the three-factor confirmatory model for the PCANC scale, Ghana and Kenya, *N* = 1988

Goodness-of Fit Statistic*	Combined (*N* = 1988)		Ghana (*N* = 996)		Kenya (*N* = 992)	
	*36 items*	*20 items*	*36 items*	*20 items*	*36 items*	*20 items*
Chi-square, *p* value	2150.827, *p* < 0.001	694.801, *p* < 0.001	2091.697, *p* < 0.001	587.204, *p* < 0.001	1343.446, *p* < 0.001	417.207, *p* < 0.001
Root mean squared error of approximation	0.048	0.054	0.067	0.069	0.050	0.055
*P* of close fit	0.922	0.050	<0.001	<0.001	0.429	0.054
Comparative fit index	0.941	0.963	0.914	0.952	0.923	0.953
Tucker–Lewis index	0.903	0.931	0.859	0.912	0.873	0.913
Standardized root mean squared residual	0.046	0.041	0.055	0.041	0.045	0.040

Model adopted is one with modification indices of a covariance of 10 or more.

*PCANC* Person-centered antenatal care.

The full 36-item scale has a Cronbach’s alpha of 0.92, increasing to 0.93 when the two wait time variables are dropped, and the 20-item scale has an alpha of 0.89. The Cronbach’s alphas for the subscales are 0.78, 0.88, and 0.78, respectively, for the “dignity and respect,” “communication and autonomy,” and “responsive and supportive care” subscales, with that for the “responsive and supportive care” subscale increasing to 0.82 when the two wait time variables are dropped. All average interitem correlations are greater than 0.2 (Supplementary Table 9). The average standardized score for the 36-item PCANC scale in the confirmation sample is 71.5 out of 100, with similar scores of 71.1 for the 34-item version and 70.6 for the 20-item version. The scores for the “dignity and respect,” “communication and autonomy,” and “responsive and supportive care” subscales are 81.2, 65.3, and 72.8, respectively, for the full sample. Scores by country are shown in Supplementary Table 9.

Cross-tabulating and regressing the full scale on patients’ ratings of satisfaction with ANC services, general perceived quality of ANC, and whether they would receive ANC in the same facility if they were to be pregnant again showed increasing PCANC scores with higher ratings of satisfaction, perceived quality of care, and intent to receive ANC in the same facility (Table 7). Scores on the 36-, 34-, and 20-item scales are strongly correlated (*r* = 1.0 between the 34- and 36-item versions and 0.97 and 0.98 with the 20-item scale), suggesting good criterion validity for the shorter versions.

**Table 7:** Crosstabulation and linear regression on the PCANC score with confirmation sample, Ghana and Kenya, *N* = 1988

	*Crosstab*			*Linear Regression*			
	*N*	Mean	SD	Coefficient	*p* value	[95% Cl]	
**Satisfaction with ANC**							
Dissatisfied	45	55.08	21.01	−15.34	0.00	−21.48	−9.21
Neither satisfied nor dissatisfied	100	52.57	13.32	−17.85	0.00	−20.58	−15.12
Satisfied	1360	70.42	15.39	Reference			
Very satisfied	483	79.98	13.50	9.55	0.00	8.10	11.01
**Perceived quality of ANC**							
Poor/very poor	36	45.81	15.03	−22.39	0.00	−27.34	−17.45
Fair	177	58.50	17.13	−9.71	0.00	−12.40	−7.01
Good	993	68.20	15.35	Reference			
Very good	616	78.04	12.15	9.84	0.00	8.48	11.19
Excellent	166	86.41	10.79	18.21	0.00	16.31	20.11
**Will return for ANC in future**							
No, never	187	69.01	17.07	−5.07	0.00	−7.63	-2.51
Yes, somewhat	304	60.32	16.95	−13.76	0.00	−15.81	-11.71
Yes, definitely	1497	74.08	15.09	Reference			

*PCANC* Person-centered antenatal care, *ANC* Antenatal care, *SD* Standard deviation, *95% CI* 95% confidence interval.

Supplementary Table 10 lists all PCANC questions, along with the reasons for their inclusion/exclusion in the scale’s final long and short forms.

## Discussion

We developed a PCANC scale with high content, construct, and criterion validity, relevant to LMICs, through a rigorous scale development process. The initial phases of scale development, including literature reviews, expert reviews, and cognitive interviews, ensured content validity—the most important form of validity for any measurement tool^48,49^. The psychometric analysis using data from a sample of pregnant and postpartum women in Ghana and Kenya yielded a 36-item comprehensive scale with adequate structural validity, comprising three subscales: “dignity and respect,” “communication and autonomy,” and “responsive and supportive care.” We also developed a shorter 20-item version for instances where the 36-item version might be too long. The full scale and subscales have good internal consistency, with Cronbach’s alpha > 0.8 for the full scale and >0.7 for the subscales. Higher scores on the scale are associated with higher satisfaction, perceived quality of care, and intent to use the facility in the future, as hypothesized, indicating criterion validity.

In general, the PCANC scale developed for LMICs performed similarly to the original PCPC-US scale^45^. The PCPC-US scale had 34 items for the full scale compared to 36 items on this scale. Most (26) items from the PCPC-US scale were retained, with nine (knocking on the door, information showing they cared, family, respected, physical abuse, discrimination, hold back on asking questions, coerced, preferred clinic, and preferred provider) excluded. These are replaced with 11 items in the current version (time to get labs and drugs, auditory privacy, attention when needed help, greeted, friendly care, received best care, communication on tests and procedures, assessment of physical wellbeing, competence, availability of washrooms, cleanliness, and enough staff) not included in the PCPC-US scale. These additional items represent issues relevant to LMICs. The same three subscales are used in both versions as they represent relevant constructs globally.

Although wait time, considered very relevant during expert reviews, performed poorly in the psychometric analyses, it has been retained in the current version of the scale. Timeliness is an important aspect of person-centered care, particularly related to responsiveness, and should be measured in any work to improve person-centered care. Including timeliness thus increases the scale’s content validity. Timeliness, however, can be considered a separate construct, as reflected in the loadings of the two wait time variables. This is also reflected in their presentation as separate domains of healthcare quality (safe, effective, patient-centered, timely, efficient, and equitable)^6^. Thus, we could have a 34-item scale with two separate timeliness items. In addition to safety, we have maintained them in one scale because they are unlikely to be measured if excluded or if each domain has a different scale. The current scale thus includes safety, patient-centeredness, and timeliness, which, when used as outcomes, can assess the effectiveness and efficiency of interventions, and when combined with equity stratifiers, can assess equitable healthcare quality.

Many of the negatively worded items (physical abuse, neglect, discrimination, and bribery) were considered relevant; however, they did not perform even moderately well, likely because of their low frequency in our sample, and were thus excluded. These items may perform better when overt mistreatment during ANC is high. Thus, we recommend assessing all 46 relevant PCANC items in future validation studies to develop the best set of items that perform well across settings. Further, many questions excluded from the scale, including accessibility, service provision, counseling, and overt mistreatment, are still relevant to high-quality PCANC. Thus, although not included in the validated PCANC scale, these should be examined separately to obtain a full picture of ANC quality.

Like prior scales, the PCANC scale includes a mix of subjective items (e.g., felt treated with respect), which expectations may influence, as well as more objective questions (e.g., procedures explained) that reflect what happens during the encounter, independent of people’s expectations^26,45^. Subjective experiences are core to person-centered care, but often hard to act on, while the more objective questions are more actionable. But both types of questions are important for comprehensively assessing person-centered care^50^. The PCANC scale is, therefore, an actionable patient-reported experience measure that can inform quality improvement activities at various levels of the health system. The full scale captures all the key domains of PCANC and can thus be used for comprehensive assessments. The subscales can be used independently, where some domains are deemed more relevant. The response format ensures that people’s responses are captured on a continuum, increasing the tool’s responsiveness to detect change. The PCANC scores can thus be tracked to examine intervention effects or to assess change over time and across settings. If changing standards and expectations are reflected in how women are treated during ANC, the tool would be useful for tracking these changes.

A potential limitation is limited generalizability due to the convenience sampling approach; however, the demographic characteristics of our sample are not too different from recent national data from the 2022 DHS in both countries^51,49^. The scale may not capture some issues relevant to other LMICs, given that the initial adaptation process was conducted only in Ghana and the final validation samples are from only Ghana and Kenya. However, based on our literature review, we believe the items will apply in most other LMIC settings as they were directly applicable in Kenya without a need for additional adaptation. What might be missing are items that are unique to different settings. For example, overt mistreatment items were excluded because of their low frequency in the study samples. They will, however, be important where overt mistreatment during ANC exists. Future testing in diverse settings is therefore needed. The most important but challenging part of tool development is ensuring content validity. Yet most validation efforts focus on just psychometric adequacy. The pool of items we developed will thus provide a starting point for future psychometric assessments in different settings. Given the rapid uptake of the PCMC scale and validation in other settings after the initial validation, this validation study in two LMIC countries will spur further validation in other LMIC settings. Other drawbacks include the scale’s length and the associated participant burden. We address this by proposing a shorter version. Like all self-reported measures, responses are prone to recall and social desirability bias, which are minimized by reducing recall time, using research assistants who are not affiliated with the facility, ensuring privacy, and assuring participants of confidentiality. The PCANC scale is grounded on a strong theoretical and empirical foundation, as it builds on prior work, including the PCMC and PCPC-US scales. The rigorous adaptation process, following standard scale development steps, ensured that the final scale had high validity and reliability and was relevant to the target population.

We present a valid and reliable comprehensive tool to measure the key domains of PCANC in LMIC contexts. Like prior person-centered tools, this tool will be valuable for needs assessment to identify gaps in PCANC to inform interventions, and for evaluations to assess the effect of interventions on PCANC. In addition, it can be used for research to examine predictors of inequities in PCANC, assess its impact on maternal and neonatal outcomes, and improve advocacy. It could also be used for routine monitoring for quality improvement and accountability. PCANC is critical to achieving the vision for maternal and newborn health. However, monitoring progress toward this vision will be difficult without measurement tools. This tool thus addresses a critical gap in efforts to measure and provide high-quality ANC in LMICs.

## Methods

We followed standard procedures for scale development, as used in the development of the PCMC and PCPC-US scales, to ensure both conceptual and psychometric adequacy.

### Setting

The initial adaptation and validation activities took place in the Upper East Region of Ghana. The final confirmation sample is the baseline data for an ongoing trial in northern Ghana’s Upper East and North East Regions and Migori and Homa Bay Counties in western Kenya. These sites have all been previously described^52,53^. The Upper East and North East Regions are neighboring regions in Ghana’s northeastern corner, both bordering Togo to the east. The Upper East Region borders Burkina Faso to the north and the North East Region to the south. The Upper East Region is divided into 15 districts, with 11 district hospitals, 67 health centers, about 419 Community-Based Health Planning and Services (CHPS) compounds, and one regional hospital that serves as a referral center for the district hospitals^54,55^. The North East is divided into six districts, with five district hospitals, 21 health centers, and 154 CHPS compounds^56^. Migori and Homabay are also neighboring counties along Lake Victoria in western Kenya. They each have eight sub-counties, each with a sub-county hospital and one county referral hospital. There are about 315 and 352 health facilities in Migori and Homa Bay, respectively, including county and sub-county hospitals, health centers, and faith-based and private health facilities^57^.

### Procedures to ensure conceptual adequacy

To ensure *conceptual adequacy*, we started with a *literature review* on women’s experiences during ANC in LMICs to update the items in the PCPC-US scale. Although the PCPC-US scale was developed following a comprehensive literature review to define the construct and its domains, the final items focused on the experiences of people of color in the US. Thus, we reviewed the literature on women’s experiences during ANC in LMICs to identify issues that are most important during ANC to pregnant people in LMICs. This included reviewing the systematic review of what matters to women during ANC, the WHO guidelines on ANC for a positive pregnancy experience, and previous publications on PCANC^1,16,58^. Following the literature review, we assessed the PCPC-US scale to identify which items may apply to ANC in LMICs. We included additional relevant items not already captured based on the literature review.

The next phase involved *expert reviews* to assess content validity: asking experts to judge the comprehensiveness of items for measuring a construct and their relevance to the target population^48,59^. Experts were purposively recruited to include maternal health experts, healthcare workers, and women with lived experiences with pregnancy (currently pregnant or had previously been pregnant). Eleven external experts (i.e., not part of our research team), which included, three maternal health researchers based in Ghana, three healthcare providers, three pregnant women, and two women who had recently given birth (recruited from health facilities in the Upper East Region of Ghana), participated in the expert reviews with our research team (four maternal health researchers, two of whom have clinical experience). This number exceeded the recommended sample size of at least six expert reviewers^60,61^. Each reviewer was first sent the initial list of questions to review individually. For each question, they were asked to think about its relevance to measuring PCANC in general (as defined for them) and to the ANC experiences of pregnant women in Ghana, and to rank it on a scale of 0 to 3 (0, not relevant; 1, somewhat relevant; 2, quite relevant; and 3, highly relevant). Experts were also asked to assess the comprehensiveness of the items for measuring PCANC on a scale of 0 to 3 (0, not comprehensive; 1, slightly comprehensive; 2, comprehensive; 3, very comprehensive) and whether all relevant aspects of PCANC were covered by these items on a scale of 0 to 2 (0, no; 1, yes, somewhat; 2, yes, definitely). In addition, they were asked to recommend what to add, remove, or change. Following the individual reviews, the same experts were brought together as a group in July 2022 for further discussion and consensus-building. This meeting was conducted in person at the Navrongo Health Research Center and lasted about four hours.

We revised the item list based on the expert reviews. Then, we conducted *cognitive interviews* with potential respondents to assess their understanding of the questions, identify problems with their wording, and determine whether the questions were relevant and context-appropriate. Trained research assistants recruited participants who had recently received ANC (pregnant or within six months of giving birth) at health facilities and conducted one-on-one interviews using the revised questions. Interviews were conducted at a convenient time and location for the participants, in English and two local languages (Gurune and Kasem), following a training session that included appropriate translations of the questions. Participants were told that their help was needed to develop the tool and that they should be comfortable recommending changes to how the questions were asked. They were then asked to respond to each question, followed by probes to understand why they answered as they did, concerns about the wording of the questions, the relevance of the questions to their care experience, and any suggestions they had for improving the questions. We used probes instead of “thinking out loud” because these have worked better in previous cognitive interviews in similar settings. Interviews were recorded, and research assistants were engaged to debrief and make needed changes after six interviews (two in each language). Six additional interviews were conducted, during which only minor changes were suggested. Twelve cognitive interviews were conducted in total in October 2022, meeting the recommended sample size of at least 10 for cognitive interviews^62,63^.

Following revisions from the cognitive interviews, the scale items were integrated into a questionnaire that included demographic and other questions, and the full questionnaire was *pretested* to identify any final issues with the scale items and the full questionnaire. We first pretested the revised tool with 12 pregnant and postpartum women, made relevant changes, and then pretested with another 12 women, which meets the recommended sample size of 15 to 30 for pretesting^64^. No significant changes were identified during the second set of pretests.

#### Survey

The final questionnaire was administered in a cross-sectional *survey* to obtain quantitative data to assess the psychometric adequacy of the scale. Eligible participants were women in the third trimester of pregnancy or who had given birth within the last six months and who received ANC during their pregnancy. The surveys were conducted with 600 women (300 prenatal and 300 postpartum) from 9^th^ August to 7^th^ September 2023. Sample size estimation was based on the rule of thumb of about 5–10 subjects per item on the scale, with 300-500 considered good and 500 or more considered very good, regardless of the number of items^65,66^. Following an analysis of data from this first survey, the questions were revised and administered as part of the baseline surveys in the CPIPE trial^53^ to 2,000 postpartum women in Ghana and Kenya (1000 per country and ~500 per region or county) from 5th March to 29th October 2024. The sample size of 2000 was based on the trial’s primary outcome (person-centered maternity care) sample size estimation. Eligible participants were postpartum women who had given birth within the 12 weeks preceding the survey in the 40 study facilities.

For both surveys, trained research assistants recruited and interviewed women in study facilities and surrounding communities. Using the delivery registers from the study facilities, the research assistants identified eligible women and contacted them for interviews. Women who had just delivered and were still in the facility, or those attending antenatal and postnatal care appointments, were recruited at the facility. Participants were informed about the study, and those who consented were invited to one-on-one interviews at a time and location convenient to them. We used a convenience sampling approach, in which all eligible women identified were interviewed until the required sample size was achieved. The surveys were programmed into REDCap, and data were entered using a tablet. All research participants at each stage provided written informed consent and received a small token of appreciation (two cakes of soap in Ghana and 400 KSH (~$3) in Kenya).

### Psychometric analysis

For the ***psychometric analysis***, we assessed the scale’s construct and criterion validity, and its internal consistency reliability^65,67,68^. We used interitem correlations and factor analysis to reduce items and assess construct validity—the degree to which the items represent the underlying conceptual structure. We first examined the distributions of the items to identify those with very low or very high frequencies and those that needed to be recoded. Negatively worded items were recoded to ensure higher numbers represent more person-centered care. Further, as in prior analyses, to ensure that all response options ranged from 0 to 3 for scoring, we took the conservative approach of treating NA responses as positive, but not perfect responses, by recoding all items that had a NA or another fourth response to the upper middle category (2: most of the time, for positively worded items, and 1: a few times, for negatively worded items)^53^. Of note, the frequency response options capture the overall ANC experience (or up to the time of the interview if still pregnant). Next, we examined the correlations between individual items to identify those with very low and very high correlations, and to determine the average interitem correlation, aiming for an ideal average interitem correlation between 0.20 and 0.40.

We then conducted iterative EFA with the initial Ghana sample, using principal factoring with oblique rotation, which allows for correlation between the rotated factors, given that the person-centered care domains are theoretically related^69^. We used the KMO measure of sampling adequacy to assess the suitability of the variables for factor analysis, aiming for values between 0.8 and 1; Kaiser’s rule of retaining only factors with eigenvalues exceeding one and the “break” in the scree plot to determine the number of factors; and factor loadings and uniqueness to assess the performance of individual items, using a cut-off of <0.3 for low loadings and >0.9 for high uniqueness to mark items for potential removal (unless there was a strong conceptual rationale for their inclusion)^65,69,70^. Internal consistency reliability was assessed using Cronbach’s alpha, aiming for values ≥ 0.7^65,67^.

Following EFA, we conducted CFA using the CPIPE baseline surveys in Kenya and Ghana to assess whether the three-factor structure could be uniformly applied across settings. In model building, we allowed error terms to correlate between items within the same proposed domain and used modification indices to systematically include the covariances of the errors. We assessed the goodness-of-fit of the full and reduced scales and subscales by estimating the RMSEA, pclose, CFI, TLI, and the SRMR^71–73^.

The responses for the final set of items are added to create summative scores, which are standardized by dividing the mean score by the maximum possible score (e.g., for a 36-item scale, the maximum score is 108 [36*3]) and multiplied by 100. This produces a standardized score ranging from zero to 100, with zero being the worst and 100 the best. Since there is no gold standard measure of PCANC, we assessed criterion validity—whether the measure is related to other measures in theoretically predictable ways—by examining how scores on the scale are associated with satisfaction and perceived quality of ANC and intent to use the same ANC facility in the future, using cross-tabulations and linear regression. We assessed the criterion validity of the short scale by examining its correlation with the full scale and these measures.

### Ethical approval

This study was conducted in accordance with all relevant ethical regulations, including the Declaration of Helsinki. Ethical approval was obtained from the Institutional Review Boards of the University of California, San Francisco (UCSF) [IRB No: 22-36140] and the Navrongo Health Research Center [IRB No: NHRCIRB460] for the validation study; and from UCSF [IRB No: 23-38843], Navrongo Health Research Center [IRB No: NHRCCIRB555], and the Kenya Medical Research Institute [IRB No: SERU/4820] for the CPIPE trial. All participants provided informed consent before participation.

## Supplementary information

Supplementary Information

## Data Availability

The datasets generated and/or analyzed during the current study are not publicly available due to the trial still ongoing but are available from the corresponding author on reasonable request.
