# Supplementary material for: Development and validation of a person-centered antenatal care scale for low- and middle-income countries
Source: NPJ Womens Health. 2026 Jun 8;4(1):29. doi: 10.1038/s44294-026-00149-4 (PMC13581594; doi:10.1038/s44294-026-00149-4)
Supplement: Supplementary file 1 [file 44294_2026_149_MOESM1_ESM.pdf]

Development and validation of a person-centered antenatal care scale for low- and middle-income countries: supplementary tables

Titles of data:

Supplementary Table 1: All PCANC questions with their original distributions before recoding in the initial validation sample

Supplementary Table 2: Exploratory factor analysis of 50 PCANC items with initial validation sample

Supplementary Table 3: Characteristics of the PCANC scale and sub-scales in the initial validation sample

Supplementary Table 4: All PCANC questions with their original distributions before recoding in the confirmation sample

Supplementary Table 5: Exploratory factor analysis of 46 PCANC items with the confirmation sample

Supplementary Table 6: Exploratory factor analysis of retained 36 PCANC items with the confirmation sample

Supplementary Table 7: Unstandardized and standardized loadings from the three-factor confirmatory model for the 36-item PCANC scale by country

Supplementary Table 8: Goodness-of-fit indicators for different three-factor confirmatory models for the PCANC scale

Supplementary Table 9: Characteristics of the PCANC scale and sub-scales in the confirmation sample

Supplementary Table 10: All PCANC questions, with final decisions and reasons for exclusion

Supplementary Table 1: All PCANC questions with their original distributions before recoding in the initial validation sample, Ghana, N=600

Question/response options	N	%
How did you feel about the amount of time you had to wait to be seen by a health worker during antenatal visits (i.e., the time from when you arrived at the health facility to when you saw the midwife or doctor)?		
It was just right	316	52.8
It was somewhat long	197	32.9
It was very long	59	9.9
It was extremely long	26	4.3
Total	598	100
How did you feel about the amount of time it took you to retrieve your folder?		
It was just right	283	47.4
It was somewhat long	102	17.1
It was very long	18	3
It was extremely long	3	0.5
Not applicable	191	32
Total	597	100
How did you feel about the amount of time it took you to get your labs done at the facility?		
It was just right	254	42.5
It was somewhat long	128	21.4
It was very long	76	12.7
It was extremely long	37	6.2
Not applicable	102	17.1
Total	597	100
How did you feel about the amount of time it took you to get your drugs from the dispensary or pharmacy?		
It was just right	403	67.4
It was somewhat long	73	12.2
It was very long	18	3
It was extremely long	5	0.8
Not applicable	97	16.2
Refused to answer	2	0.3
Total	598	100
How did you feel about the amount of time the doctor or midwife spent with you?		
It was just right	517	86.5
It was too long	24	4
It was somewhat short	51	8.5
It was very short	6	1
Total	598	100
Did the health workers introduce themselves to you when they first saw you? (If you were seen by only one health worker and they introduced themselves, you can select yes, all of them)		
No, none of them	263	44.1
Yes, a few of them	97	16.2
Yes, most of them	61	10.2
Yes, all of them	175	29.3
Refused to answer	1	0.2
Total	597	100
Did they greet you well when you arrived?		
No, none of them	81	13.6
Yes, a few of them	128	21.4
Yes, most of them	82	13.7
Yes, all of them	306	51.3
Total	597	100
Did they call you by your name (or appropriately)?		
No, never	93	15.6
Yes, a few times	87	14.6
Yes, most of the time	96	16.1
Yes, all the time	320	53.7
Total	596	100
Did they treat you with respect?		

No, never	2	0.3
Yes, a few times	27	4.5
Yes, most of the time	78	13.1
Yes, all the time	489	81.9
Refused to answer	1	0.2
Total	597	100
Were they nice to you?		
No, none of them	1	0.2
Yes, a few of them	34	5.7
Yes, most of them	105	17.6
Yes, all of them	456	76.4
Refused to answer	1	0.2
Total	597	100
Did you feel your knowledge was valued?<br>*If need to clarify:* Did they appreciate or accept your ideas or knowledge about your health?		
No, never	5	0.8
Yes, a few times	30	5
Yes, most of the time	117	19.6
Yes, all the time	443	74.2
Don't know	2	0.3
Total	597	100
Did you feel heard by the health workers?		
No, never	6	1
Yes, a few times	30	5
Yes, most of the time	114	19.1
Yes, all the time	447	74.9
Total	597	100
During physical exams (like abdominal and pelvic exams) were you covered up with a cloth or blanket or screened with a curtain so that you did not feel exposed?		
No, never	33	5.5
Yes, a few times	10	1.7
Yes, most of the time	34	5.7
Yes, all the time	515	86.3
Not applicable	5	0.8
Total	597	100
Did you feel your health information was kept confidential by the health workers?		
No, never	52	8.7
Yes, a few times	22	3.7
Yes, most of the time	116	19.4
Yes, all the time	402	67.3
Refused to answer	5	0.8
Total	597	100
Did you feel you could discuss your problems with the health workers?		
No, never	62	10.4
Yes, a few times	29	4.9
Yes, most of the time	67	11.2
Yes, all the time	438	73.4
Refused to answer	1	0.2
Total	597	100
Did the health workers involve you in decisions about your care?		
No, never	55	9.2
Yes, a few times	24	4
Yes, most of the time	100	16.8
Yes, all the time	417	69.8
Refused to answer	1	0.2
Total	597	100
Did the health workers ask your permission before examining or doing procedures on you?		
No, never	5	0.8
Yes, a few times	12	2
Yes, most of the time	58	9.7
Yes, all the time	521	87.3

Refused to answer	1	0.2
Total	597	100
Did they explain to you why they were doing examinations or procedures on you?		
No, never	35	5.9
Yes, a few times	15	2.5
Yes, most of the time	74	12.4
Yes, all the time	472	79.2
Total	596	100
Did you feel you understood the purpose of any tests or procedures you were asked to do? (*Clarify*: like urine or blood tests, ultrasound, etc., that you were asked to do including those you were asked to do outside the facility)		
No, never	21	3.5
Yes, a few times	21	3.5
Yes, most of the time	65	10.9
Yes, all the time	486	81.4
Not applicable	2	0.3
Refused to answer	2	0.3
Total	597	100
Did they explain to you why they were giving or prescribing you any medicine?		
No, never	29	4.9
Yes, a few times	24	4
Yes, most of the time	65	10.9
Yes, all the time	461	77.2
Not applicable/I was not given any medicine	17	2.8
Refused to answer	1	0.2
Total	597	100
Did you feel you understood the purpose of any medicines you were given or prescribed to go and buy?		
No, never	26	4.4
Yes, a few times	21	3.5
Yes, most of the time	60	10.1
Yes, all the time	484	81.1
Not applicable	6	1
Total	597	100
Did you feel you could ask the health workers any questions you had?		
No, never	10	1.7
Yes, a few times	22	3.7
Yes, most of the time	63	10.6
Yes, all the time	502	84.1
Total	597	100
Did you hold back from asking questions for any reason?		
No, never	510	85.3
Yes, a few times	41	6.9
Yes, most of the time	9	1.5
Yes, all the time	5	0.8
Not applicable/I did not have any questions	33	5.5
Total	598	100
Did the health workers at the facility ask you if you had any questions?		
No, never	58	9.7
Yes, a few times	42	7
Yes, most of the time	104	17.4
Yes, all the time	393	65.8
Total	597	100
Did they encourage you to ask questions?		
No, never	61	10.2
Yes, a few times	51	8.6
Yes, most of the time	115	19.3
Yes, all the time	369	61.9
Total	596	100
Do you feel your questions were answered when you asked them?		
Yes, a few times	24	4

Yes, most of the time	75	12.6
Yes, all the time	409	68.5
Not applicable/I did not ask any questions	89	14.9
Total	597	100
Did they check that you understood information that was given to you?		
No, never	51	8.5
Yes, a few times	44	7.4
Yes, most of the time	107	17.9
Yes, all the time	395	66.2
Total	597	100
Did the health workers speak to you in a language you could understand or using language you could understand?		
No, none of them	3	0.5
Yes, a few of them	14	2.3
Yes, most of them	74	12.4
Yes, all of them	506	84.8
Total	597	100
Did they speak to you using words you could understand?		
No, never	26	4.4
Yes, a few times	26	4.4
Yes, most of the time	90	15.1
Yes, all the time	454	76.2
Total	596	100
Did you feel the health workers cared about you?		
Yes, a few times	17	2.9
Yes, most of the time	98	16.4
Yes, all the time	481	80.7
Total	596	100
Did you feel they took the best care of you?		
Yes, a few times	22	3.7
Yes, most of the time	99	16.6
Yes, all the time	475	79.6
Refused to answer	1	0.2
Total	597	100
Did you feel forced into a decision by health workers?		
No, never	533	89.1
Yes, a few times	16	2.7
Yes, most of the time	11	1.8
Yes, all the time	37	6.2
Refused to answer	1	0.2
Total	598	100
Did a health worker discuss your birth plan with you (e.g., where you wanted to give birth or what you wanted during the birth?)		
No, never	162	27.1
Yes, once	77	12.9
Yes, a few times	135	22.6
Yes, many times	222	37.2
Refused to answer	1	0.2
Total	597	100
Did they ask you about your physical health? [Local translation: Did they ask you how you were feeling in your body?]		
No, never	27	4.5
Yes, a few times	24	4
Yes, most of the time	81	13.6
Yes, all the time	465	77.9
Total	597	100
Did they give you the support to deal with your mental and emotional health? [Local translation: Did they give you the support to deal with how you were feeling in your mind or heart if you needed it?]		
No, never	185	31
Yes, a few times	66	11.1

Yes, most of the time	77	12.9
Yes, all the time	269	45.1
Total	597	100
Did they ask you about your mood? [Local translation: Did they ask you how you were feeling in your heart?]		
No, never	203	34
Yes, a few times	80	13.4
Yes, most of the time	86	14.4
Yes, all the time	227	38
Refused to answer	1	0.2
Total	597	100
Did they give you the support to deal with your mental and emotional health? [Local translation: Did they give you the support to deal with how you were feeling in your mind or heart if you needed it?]		
No, never	49	8.2
Yes, a few times	58	9.7
Yes, most of the time	110	18.4
Yes, all the time	221	37
Not applicable/I did not need emotional support	159	26.6
Total	597	100
Did the health workers respect your family or companions who were with you?		
No, never	8	1.3
Yes, a few times	16	2.7
Yes, most of the time	51	8.5
Yes, all the time	240	40.2
Not applicable/I did not have family or companions present	282	47.2
Total	597	100
When you needed support from the providers with regards to your health, did they pay attention?		
No, never	13	2.2
Yes, a few times	18	3
Yes, most of the time	92	15.4
Yes, all the time	365	61.1
Not applicable/I did not need help	108	18.1
Refused to answer	1	0.2
Total	597	100
Did you feel the health workers avoided, ignored, or neglected you?		
No, never	562	94
Yes, once	6	1
Yes, a few times	12	2
Yes, many times	18	3
Total	598	100
Did you feel they talked to you badly (For example, shouted at you, scolded, insulted, or threatened, you)?		
No, never	566	94.6
Yes, once	13	2.2
Yes, a few times	13	2.2
Yes, many times	5	0.8
Refused to answer	1	0.2
Total	598	100
Did you feel they handled you badly (For example pushed, beat, slapped, pinched or physically restrained you)?		
No, never	594	99.3
Yes, once	1	0.2
Yes, a few times	2	0.3
Yes, many times	1	0.2
Total	598	100
Did you feel you could trust the health workers with regards to your care?		
No, never	16	2.7
Yes, a few times	44	7.4
Yes, most of the time	131	21.9
Yes, all the time	406	68

Total	597	100
Did you feel the health workers were competent?		
No, none of them	1	0.2
Yes, a few of them	51	8.5
Yes, most of them	152	25.5
Yes, all of them	390	65.3
Don't know	3	0.5
Total	597	100
Did you feel discriminated against by the health workers in any way? I.e., did you feel you were treated differently based on something about you?		
No, never	571	95.5
Yes, a few times	18	3
Yes, most of the time	7	1.2
Yes, all the time	2	0.3
Total	598	100
Did you feel the health facility environment, including the washrooms were clean?		
No, never	43	7.2
Yes, a few times	49	8.2
Yes, most of the time	136	22.8
Yes, all the time	336	56.3
No washroom available	32	5.4
Refused to answer	1	0.2
Total	597	100
In general, did you feel physically safe in the place you received antenatal care?		
No, never	10	1.7
Yes, a few times	31	5.2
Yes, most of the time	135	22.6
Yes, all the time	420	70.4
Refused to answer	1	0.2
Total	597	100
Do you think there was enough health staff in the facility to care for you?		
No, never	27	4.5
Yes, a few times	80	13.4
Yes, most of the time	159	26.6
Yes, all the time	331	55.4
Total	597	100
Were you able to go to your preferred place/clinic for antenatal care?		
No, never	26	4.4
Yes, a few times	9	1.5
Yes, most of the time	48	8
Yes, all the time	421	70.5
Not applicable/I did not have a preferred place/clinic	93	15.6
Total	597	100
Were you able to see your preferred provider for antenatal care?		
No, never	18	3
Yes, a few times	35	5.9
Yes, most of the time	93	15.6
Yes, all the time	294	49.2
Not applicable/I did not have a preferred provider	157	26.3
Total	597	100
During your antenatal care, did any health worker at the facility ask you or your family for an unofficial payment?		
No, never	575	96.2
Yes, a few times	17	2.8
Yes, most of the time	2	0.3
Yes, all the time	3	0.5
Refused to answer	1	0.2
Total	598	100
**Global ratings for criterion validity**		
In general, how satisfied were you with how you were treated by the healthcare workers during your antenatal care?		

Dissatisfied	5	0.8
Neither satisfied nor dissatisfied	19	3.2
Satisfied	284	47.6
Very satisfied	289	48.4
Total	597	100
In general, how would you rate the quality of care you received during delivery?		
Very Poor	2	0.3
Poor	9	1.5
Fair	20	3.4
Good	183	30.7
Very Good	237	39.7
Excellent	144	24.1
Refused to answer	2	0.3
Total	597	100
Will you want to give birth in the place you received antenatal care? *For postpartum respondents*: If you were to have another baby, will you want to give birth in the place you received antenatal care?		
No, never	63	10.6
Yes, somewhat	56	9.4
Yes, definitely	477	79.9
Refused to answer	1	0.2
Total	597	100

Notes: PCANC: Person-centered antenatal care

Supplementary Table 2: Exploratory factor analysis of 50 PCANC items with initial validation sample, Ghana, N=595

	3 factor structure				*Single factor structure*	
Label	Factor1	Factor2	Factor3	Uniqueness	Factor1	Uniqueness
Wait time for provider	0.11			0.99		0.99
Wait time for labs or drugs				0.98		0.99
Time with provider	0.29			0.92	0.19	0.96
Introductions by provider	0.32			0.79	0.45	0.79
Greeted	0.62			0.64	0.53	0.72
Called preferred name	0.39			0.87	0.31	0.90
Treat you with respect	0.66			0.52	0.65	0.58
Friendly care	0.67			0.51	0.65	0.57
Knowledge valued	0.56			0.56	0.64	0.59
Heard and listened to	0.60			0.63	0.57	0.68
Privacy/not exposed	0.22			0.93	0.22	0.95
Info confidentiality	0.36			0.77	0.40	0.84
Auditory privacy	0.58			0.69	0.48	0.77
Involved in decisions	0.35			0.75	0.48	0.77
Consent	0.50			0.61	0.57	0.68
Explain exams/procedures	0.31		0.36	0.65	0.56	0.69
Understood tests	0.32		0.52	0.52	0.60	0.64
Explain medications			0.81	0.35	0.56	0.69
Understood medicines			0.79	0.38	0.54	0.71
Told examination results		0.37		0.87	0.19	0.96
Told test results		0.36		0.87	0.16	0.98
Could ask any questions			0.27	0.76	0.47	0.78
Hold back questions		0.32		0.91	0.15	0.98
Asked if questions		0.32	0.39	0.59	0.56	0.69
Encouraged questions		0.40	0.43	0.50	0.59	0.65
Questions were answered		0.38		0.64	0.57	0.68
Check understood information	0.32	0.33		0.61	0.61	0.63
Language they understood	0.49			0.82	0.31	0.90
Language level they understood	0.29			0.87	0.35	0.88
Showed they cared	0.50			0.63	0.60	0.64
Received best care	0.56			0.57	0.64	0.59
Pressured		0.27		0.91		0.99
Asked about birth plan		0.28		0.88	0.25	0.94
Physical wellbeing assessed		0.26		0.76	0.47	0.78
Mental/emotions wellbeing assessed		0.70		0.57	0.38	0.85
Resources for emotional/mental wellbeing		0.66		0.59	0.41	0.83
Family respected		0.27		0.88	0.29	0.92
Attention when needed help		0.37		0.77	0.43	0.82
Neglected			0.10	0.97	0.17	0.97
Verbal abuse	0.18			0.90	0.32	0.90
Physical abuse				1.00		1.00
Trust	0.53			0.66	0.56	0.69
Competence	0.67			0.53	0.63	0.60
Discrimination	0.14			0.95	0.21	0.95
Cleanliness	0.28			0.93	0.18	0.97
Safe	0.46			0.81	0.37	0.87
Enough staff		0.21		0.90	0.23	0.95
Preferred clinic		0.22		0.89	0.27	0.93
Preferred provider		0.44		0.71	0.43	0.82
Bribe		0.10		0.99		1.00

Notes: PCANC: Person-centered antenatal care. Blanks imply less than 0.1

Supplementary Table 3: Characteristics of the PCANC scale and sub-scales in the initial validation sample, Ghana, N=595

		Internal Consistency		*Standardized scores*			
	Number of items	Average interitem correlation	Cronbach's alpha	Mean	Std. dev.	Min	Max
Full 36-item PCANC scale	36	0.18	0.89	77.57	11.87	22.22	96.30
Dignity and Respect subscale	8	0.27	0.74	86.44	14.99	16.67	100
Communication and autonomy subscale	15	0.20	0.79	73.49	14.96	15.56	100
Responsive and supportive care subscale	13	0.15	0.70	76.82	11.59	28.21	100

Notes: PCANC: Person-centered antenatal care

Supplementary Table 4: All PCANC questions with their original distributions before recoding in the confirmation sample, Ghana and Kenya, N=1,992

	Kenya		Ghana		Total	
	N	%	*N*	%	*N*	%
How did you feel about the amount of time you had to wait to be seen by a health worker during antenatal visits (i.e., the time from when you arrived at the health facility to when you saw the midwife or doctor)?						
It was just right	618	62.1	578	58	1,196	60
It was somewhat long	244	24.5	294	29.5	538	27
It was very long	115	11.6	116	11.6	231	11.6
It was extremely long	18	1.8	9	0.9	27	1.4
Total	995	100	997	100	1,992	100
How did you feel about the amount of time it took you to get your labs done at the facility?						
It was just right	573	57.6	477	47.8	1,050	52.7
It was somewhat long	234	23.5	224	22.5	458	23
It was very long	149	15	177	17.8	326	16.4
It was extremely long	27	2.7	111	11.1	138	6.9
Not applicable	12	1.2	8	0.8	20	1
Total	995	100	997	100	1,992	100
How did you feel about the amount of time the doctor or midwife spent with you?						
It was just right	881	88.5	861	86.4	1,742	87.4
It was too long	58	5.8	65	6.5	123	6.2
It was somewhat short	47	4.7	59	5.9	106	5.3
It was very short	9	0.9	12	1.2	21	1.1
Total	995	100	997	100	1,992	100
Did the health workers introduce themselves to you when they first saw you? (If you were seen by only one health worker and they introduced themselves, you can select yes, all of them)						
No, none of them	687	69.1	486	48.7	1,173	58.9
Yes, a few of them	158	15.9	196	19.7	354	17.8
Yes, most of them	58	5.8	194	19.5	252	12.7
Yes, all of them	88	8.9	121	12.1	209	10.5
Refused to answer	3	0.3	0	0	3	0.2
Total	994	100	997	100	1,991	100
Did they greet you well when you arrived or responded well if you greeted first?						
No, none of them	64	6.4	51	5.1	115	5.8
Yes, a few of them	235	23.6	124	12.4	359	18
Yes, most of them	229	23	281	28.2	510	25.6
Yes, all of them	466	46.8	540	54.2	1,006	50.5
Refused to answer	1	0.1	1	0.1	2	0.1
Total	995	100	997	100	1,992	100
Did they call you by your name (or appropriately)?						
No, never	87	8.7	133	13.3	220	11
Yes, a few times	110	11.1	111	11.1	221	11.1
Yes, most of the time	185	18.6	280	28.1	465	23.3
Yes, all the time	613	61.6	472	47.3	1,085	54.5
N/a-don't know	0	0	1	0.1	1	0.1
Total	995	100	997	100	1,992	100
Did they treat you with respect?						
No, never	5	0.5	6	0.6	11	0.6
Yes, a few times	85	8.5	59	5.9	144	7.2
Yes, most of the time	201	20.2	262	26.3	463	23.2

Yes, all the time	704	70.8	668	67	1,372	68.9
N/a-don't know	0	0	2	0.2	2	0.1
Total	995	100	997	100	1,992	100
Were they friendly or nice to you?						
No, never	14	1.4	9	0.9	23	1.2
Yes, a few times	91	9.1	72	7.2	163	8.2
Yes, most of the time	204	20.5	253	25.4	457	22.9
Yes, all the time	686	68.9	661	66.3	1,347	67.6
N/a-don't know	0	0	2	0.2	2	0.1
Total	995	100	997	100	1,992	100
Did the health workers ask your permission before examining or doing procedures on you?						
No, never	272	27.3	15	1.5	287	14.4
Yes, a few times	182	18.3	48	4.8	230	11.5
Yes, most of the time	159	16	240	24.1	399	20
Yes, all the time	379	38.1	693	69.5	1,072	53.8
N/a-don't know	3	0.3	1	0.1	4	0.2
Total	995	100	997	100	1,992	100
D During physical exams (like abdominal and pelvic exams) were you covered up with a cloth or blanket or screened with a curtain so that you did not feel exposed?						
No, never	152	15.3	55	5.5	207	10.4
Yes, a few times	89	8.9	31	3.1	120	6
Yes, most of the time	162	16.3	150	15.1	312	15.7
Yes, all the time	587	59	758	76.1	1,345	67.6
N/a-don't know	5	0.5	2	0.2	7	0.4
Total	995	100	996	100	1,991	100
Did you feel your knowledge was valued? If need to clarify: Did they appreciate or accept your ideas or knowledge about your health and your baby/ies's health?						
No, never	46	4.6	33	3.3	79	4
Yes, a few times	164	16.5	174	17.5	338	17
Yes, most of the time	234	23.5	306	30.7	540	27.1
Yes, all the time	377	37.9	467	46.8	844	42.4
N/a-don't know	174	17.5	17	1.7	191	9.6
Total	995	100	997	100	1,992	100
Did you feel heard by the health workers?						
No, never	37	3.7	80	8	117	5.9
Yes, a few times	133	13.4	140	14	273	13.7
Yes, most of the time	224	22.5	267	26.8	491	24.6
Yes, all the time	461	46.3	502	50.4	963	48.3
N/a-don't know	140	14.1	8	0.8	148	7.4
Total	995	100	997	100	1,992	100
Did you feel your health information was kept confidential by the health workers?						
No, never	23	2.3	33	3.3	56	2.8
Yes, a few times	47	4.7	78	7.8	125	6.3
Yes, most of the time	214	21.5	227	22.8	441	22.1
Yes, all the time	644	64.7	614	61.6	1,258	63.2
N/a-don't know	67	6.7	45	4.5	112	5.6
Total	995	100	997	100	1,992	100
Did you feel you could discuss your problems with the health workers, without others not involved in your care overhearing your conversations?						
No, never	179	18	119	11.9	298	15

Yes, a few times	102	10.3	104	10.4	206	10.3
Yes, most of the time	183	18.4	259	26	442	22.2
Yes, all the time	515	51.8	509	51.1	1,024	51.4
N/a-don’t know	16	1.6	5	0.5	21	1.1
Total	995	100	996	100	1,991	100
Did they explain to you why they were doing examinations or tests on you?						
No, never	261	26.2	98	9.8	359	18
Yes, a few times	180	18.1	103	10.3	283	14.2
Yes, most of the time	200	20.1	250	25.1	450	22.6
Yes, all the time	350	35.2	545	54.7	895	44.9
N/a-don’t know	4	0.4	1	0.1	5	0.3
Total	995	100	997	100	1,992	100
Did the health workers involved you in decisions about your care?						
No, never	189	19	66	6.6	255	12.8
Yes, a few times	183	18.4	145	14.6	328	16.5
Yes, most of the time	275	27.6	269	27	544	27.3
Yes, all the time	330	33.2	514	51.6	844	42.4
N/a-don’t know	18	1.8	2	0.2	20	1
Total	995	100	996	100	1,991	100
Did you feel you understood the purpose of any tests or procedures you were asked to do? (*Clarify*: like urine or blood tests, ultrasound, etc., that you were asked to do including those you were asked to do outside the facility)						
No, never	232	23.3	88	8.8	320	16.1
Yes, a few times	227	22.8	129	12.9	356	17.9
Yes, most of the time	190	19.1	274	27.5	464	23.3
Yes, all the time	336	33.8	503	50.5	839	42.1
N/a-don’t know	10	1	3	0.3	13	0.7
Total	995	100	997	100	1,992	100
Was your blood pressure taken? If YES, was your blood pressure taken all the time, most of the time, or a few times?						
No, never	251	25.2	111	11.1	362	18.2
Yes, a few times	152	15.3	130	13	282	14.2
Yes, most of the time	173	17.4	248	24.9	421	21.1
Yes, all the time	413	41.5	507	50.9	920	46.2
N/a-don’t know	5	0.5	1	0.1	6	0.3
Refused to answer	1	0.1	0	0	1	0.1
Total	995	100	997	100	1,992	100
Did they tell you the results of tests you did like blood or urine tests?						
No, never	288	28.9	108	10.8	396	19.9
Yes, a few times	205	20.6	159	15.9	364	18.3
Yes, most of the time	153	15.4	249	25	402	20.2
Yes, all the time	334	33.6	480	48.1	814	40.9
N/a-don’t know	14	1.4	1	0.1	15	0.8
Refused to answer	1	0.1	0	0	1	0.1
Total	995	100	997	100	1,992	100
Did they explain to you why they were giving or prescribing you any medicine?						
No, never	154	15.5	88	8.8	242	12.1
Yes, a few times	195	19.6	118	11.8	313	15.7
Yes, most of the time	197	19.8	245	24.6	442	22.2
Yes, all the time	438	44	544	54.6	982	49.3
N/a-don’t know	11	1.1	2	0.2	13	0.7
Total	995	100	997	100	1,992	100

Did you understand the purpose of any medicines you were given or prescribed?						
No, never	148	14.9	81	8.1	229	11.5
Yes, a few times	206	20.7	135	13.5	341	17.1
Yes, most of the time	205	20.6	255	25.6	460	23.1
Yes, all the time	424	42.6	522	52.4	946	47.5
N/a-don't know	11	1.1	4	0.4	15	0.8
Refused to answer	1	0.1	0	0	1	0.1
Total	995	100	997	100	1,992	100
Did you feel you could ask the health workers any questions you had?						
No, never	100	10.1	72	7.2	172	8.6
Yes, a few times	201	20.2	147	14.7	348	17.5
Yes, most of the time	181	18.2	268	26.9	449	22.5
Yes, all the time	494	49.6	508	51	1,002	50.3
N/a-don't know	19	1.9	2	0.2	21	1.1
Total	995	100	997	100	1,992	100
Did you hold back from asking questions for any reason?						
No, never	714	71.8	694	69.6	1,408	70.7
Yes, a few times	150	15.1	104	10.4	254	12.8
Yes, most of the time	61	6.1	104	10.4	165	8.3
Yes, all the time	57	5.7	86	8.6	143	7.2
N/a-don't know	13	1.3	9	0.9	22	1.1
Total	995	100	997	100	1,992	100
Did they encourage you to ask questions?						
No, never	276	27.7	131	13.1	407	20.4
Yes, a few times	203	20.4	149	14.9	352	17.7
Yes, most of the time	161	16.2	258	25.9	419	21
Yes, all the time	350	35.2	457	45.8	807	40.5
N/a-don't know	5	0.5	2	0.2	7	0.4
Total	995	100	997	100	1,992	100
Do you feel your questions were answered when you asked them?						
No, never	44	4.4	77	7.7	121	6.1
Yes, a few times	156	15.7	154	15.4	310	15.6
Yes, most of the time	219	22	242	24.3	461	23.1
Yes, all the time	448	45	504	50.6	952	47.8
N/a-don't know	127	12.8	20	2	147	7.4
Refused to answer	1	0.1	0	0	1	0.1
Total	995	100	997	100	1,992	100
Did they check that you understood information that was given to you?						
No, never	259	26	87	8.7	346	17.4
Yes, a few times	202	20.3	190	19.1	392	19.7
Yes, most of the time	167	16.8	265	26.6	432	21.7
Yes, all the time	315	31.7	446	44.7	761	38.2
N/a-don't know	52	5.2	9	0.9	61	3.1
Total	995	100	997	100	1,992	100
Did you feel you understood the purpose of any medicines you were given or prescribed for yourself?						
No, none of them	2	0.2	4	0.4	6	0.3
Yes, a few of them	7	0.7	48	4.8	55	2.8
Yes, most of them	83	8.3	217	21.8	300	15.1
Yes, all of them	903	90.8	726	72.9	1,629	81.8
Refused to answer	0	0	1	0.1	1	0.1

Total	995	100	996	100	1,991	100
Did you feel the health workers cared about you?						
No, never	9	0.9	7	0.7	16	0.8
Yes, a few times	79	7.9	87	8.7	166	8.3
Yes, most of the time	280	28.1	301	30.2	581	29.2
Yes, all the time	624	62.7	601	60.3	1,225	61.5
Refused to answer	3	0.3	0	0	3	0.2
Total	995	100	996	100	1,991	100
Did you feel they took the best care of you?						
No, never	10	1	9	0.9	19	1
Yes, a few times	78	7.8	84	8.4	162	8.1
Yes, most of the time	306	30.8	312	31.3	618	31
Yes, all the time	601	60.4	591	59.3	1,192	59.8
Refused to answer	0	0	1	0.1	1	0.1
Total	995	100	997	100	1,992	100
Did you feel forced into a decision by health workers?						
No, never	885	88.9	910	91.3	1,795	90.1
Yes, a few times	46	4.6	26	2.6	72	3.6
Yes, most of the time	24	2.4	22	2.2	46	2.3
Yes, all the time	40	4	39	3.9	79	4
Total	995	100	997	100	1,992	100
Did a health worker discuss your birth plan with you (e.g., where you wanted to give birth or what you wanted during the birth?)						
No, never	325	32.7	355	35.6	680	34.1
Yes, once	257	25.8	103	10.3	360	18.1
Yes, a few times	155	15.6	241	24.2	396	19.9
Yes, many times	258	25.9	296	29.7	554	27.8
Refused to answer	0	0	2	0.2	2	0.1
Total	995	100	997	100	1,992	100
Did they ask you about your physical health? [Local translation: Did they ask you how you were feeling in your body?]						
No, never	137	13.8	36	3.6	173	8.7
Yes, a few times	160	16.1	106	10.6	266	13.4
Yes, most of the time	236	23.7	227	22.8	463	23.2
Yes, all the time	460	46.2	628	63	1,088	54.6
N/a-don’t know	2	0.2	0	0	2	0.1
Total	995	100	997	100	1,992	100
Did they ask you about your mental health? [Local translation: Did they ask you how you were feeling in your mind?]						
No, never	564	56.7	335	33.6	899	45.1
Yes, a few times	173	17.4	201	20.2	374	18.8
Yes, most of the time	90	9	167	16.8	257	12.9
Yes, all the time	161	16.2	283	28.4	444	22.3
N/a-don’t know	7	0.7	11	1.1	18	0.9
Total	995	100	997	100	1,992	100
Did they give you the support to deal with your mental and emotional health? [Local translation: Did they give you the support to deal with how you were feeling in your mind or heart if you needed it?]						
No, never	496	49.8	340	34.1	836	42
Yes, a few times	128	12.9	146	14.6	274	13.8
Yes, most of the time	64	6.4	156	15.6	220	11

Yes, all the time	107	10.8	249	25	356	17.9
N/a-don't know	200	20.1	106	10.6	306	15.4
Total	995	100	997	100	1,992	100
Did the health workers respect your family or companions who were with you?						
No, never	22	2.2	19	1.9	41	2.1
Yes, a few times	70	7	52	5.2	122	6.1
Yes, most of the time	119	12	240	24.1	359	18
Yes, all the time	312	31.4	524	52.6	836	42
N/a-don't know	471	47.3	162	16.2	633	31.8
Refused to answer	1	0.1	0	0	1	0.1
Total	995	100	997	100	1,992	100
When you needed support from the providers with regards to your health, did they pay attention?						
No, never	17	1.7	10	1	27	1.4
Yes, a few times	113	11.4	110	11	223	11.2
Yes, most of the time	256	25.7	293	29.4	549	27.6
Yes, all the time	505	50.8	578	58	1,083	54.4
N/a-don't know	103	10.4	6	0.6	109	5.5
Refused to answer	1	0.1	0	0	1	0.1
Total	995	100	997	100	1,992	100
Did you feel you could trust the health workers with regards to your care?						
No, never	18	1.8	18	1.8	36	1.8
Yes, a few times	87	8.7	104	10.4	191	9.6
Yes, most of the time	285	28.6	312	31.3	597	30
Yes, all the time	603	60.6	559	56.1	1,162	58.3
N/a-don't know	2	0.2	4	0.4	6	0.3
Total	995	100	997	100	1,992	100
Did you feel discriminated against by the health workers in any way? I.e., did you feel you were treated differently based on something about you?						
No, never	940	94.5	904	90.7	1,844	92.6
Yes, a few times	36	3.6	44	4.4	80	4
Yes, most of the time	8	0.8	24	2.4	32	1.6
Yes, all the time	8	0.8	16	1.6	24	1.2
N/a-don't know	3	0.3	9	0.9	12	0.6
Total	995	100	997	100	1,992	100
Did you feel the health workers avoided, ignored, or neglected you?						
No, never	945	95	960	96.3	1,905	95.6
Yes, once	33	3.3	23	2.3	56	2.8
Yes, a few times	13	1.3	13	1.3	26	1.3
Yes, many times	4	0.4	1	0.1	5	0.3
Total	995	100	997	100	1,992	100
Did you feel they talked to you badly (For example, shouted at you, scolded, insulted, or threatened, you)?						
No, never	939	94.4	934	93.7	1,873	94
Yes, once	34	3.4	28	2.8	62	3.1
Yes, a few times	20	2	29	2.9	49	2.5
Yes, many times	2	0.2	6	0.6	8	0.4
Total	995	100	997	100	1,992	100
Did you feel the health workers were competent?						
No, none of them	13	1.3	6	0.6	19	1
Yes, a few of them	43	4.3	95	9.5	138	6.9

Yes, most of them	232	23.3	349	35	581	29.2
Yes, all of them	696	69.9	541	54.3	1,237	62.1
Don't know	11	1.1	6	0.6	17	0.9
Total	995	100	997	100	1,992	100
Could you use the washrooms in the facility if you needed to?						
No, never	36	3.6	106	10.6	142	7.1
Yes, a few times	180	18.1	156	15.7	336	16.9
Yes, most of the time	125	12.6	223	22.4	348	17.5
Yes, all the time	625	62.8	485	48.7	1,110	55.8
N/a-don't know	29	2.9	26	2.6	55	2.8
Total	995	100	996	100	1,991	100
Did you feel the health facility environment, including the washrooms were clean?						
No, never	70	7	61	6.1	131	6.6
Yes, a few times	178	17.9	137	13.7	315	15.8
Yes, most of the time	239	24	339	34	578	29
Yes, all the time	497	49.9	443	44.4	940	47.2
N/a-don't know	11	1.1	17	1.7	28	1.4
Total	995	100	997	100	1,992	100
In general, did you feel safe in the health facility?						
No, never	7	0.7	32	3.2	39	2
Yes, a few times	57	5.7	81	8.1	138	6.9
Yes, most of the time	243	24.4	295	29.6	538	27
Yes, all the time	686	68.9	587	58.9	1,273	63.9
N/a-don't know	1	0.1	2	0.2	3	0.2
Refused to answer	1	0.1	0	0	1	0.1
Total	995	100	997	100	1,992	100
Do you think there was enough health staff in the facility to care for you?						
No, never	109	11	51	5.1	160	8
Yes, a few times	106	10.7	109	10.9	215	10.8
Yes, most of the time	239	24	279	28	518	26
Yes, all the time	533	53.6	557	55.9	1,090	54.7
N/a-don't know	8	0.8	1	0.1	9	0.5
Total	995	100	997	100	1,992	100
During your antenatal care, did any health worker at the facility ask you or your family for an unofficial payment?						
No, never	875	87.9	792	79.4	1,667	83.7
Yes, a few times	89	8.9	92	9.2	181	9.1
Yes, most of the time	12	1.2	39	3.9	51	2.6
Yes, all the time	15	1.5	52	5.2	67	3.4
N/a-don't know	4	0.4	21	2.1	25	1.3
Refused to answer	0	0	1	0.1	1	0.1
Total	995	100	997	100	1,992	100

Notes: PCANC: Person-centered antenatal care

Supplementary Table 5: Exploratory factor analysis of 46 PCANC items with the confirmation sample, Ghana and Kenya, N=1,988

	3 factor structure				*Single factor structure*	
Item	Factor1	Factor2	Factor3	Uniqueness	Factor1	Uniqueness
Introductions by provider	0.41			0.82	0.33	0.89
Greeted		0.47		0.63	0.58	0.66
Called preferred name		0.44		0.83	0.34	0.88
Treat you with respect		0.74		0.44	0.65	0.58
Friendly care		0.74		0.43	0.65	0.57
Consent	0.53			0.67	0.53	0.72
Privacy not exposed	0.31			0.74	0.50	0.75
Knowledge valued	0.30	0.50		0.49	0.71	0.50
Heard and listened to		0.52		0.56	0.63	0.60
Info confidentiality		0.49		0.74	0.45	0.80
Auditory privacy	0.25	0.24		0.80	0.43	0.81
Explain exams/procedures	0.81			0.41	0.65	0.58
Involved in decisions	0.55			0.52	0.67	0.55
Understood tests	0.81			0.39	0.67	0.55
Told examination results	0.71			0.54	0.56	0.69
Told test results	0.76			0.50	0.58	0.66
Explain medications	0.81			0.36	0.70	0.51
Understood medicines	0.80			0.38	0.69	0.52
Could ask any questions	0.40			0.57	0.66	0.57
Encouraged questions	0.64			0.47	0.69	0.53
Questions were answered	0.42			0.56	0.66	0.56
Check understood information	0.62			0.47	0.70	0.51
language they understood		0.53		0.77	0.28	0.92
Showed they cared		0.82		0.34	0.70	0.50
Received best care		0.78		0.37	0.70	0.51
Asked about birth plan	0.42			0.77	0.40	0.84
Physical wellbeing assessed	0.39			0.76	0.47	0.78
Mental/emotions wellbeing assessed	0.62			0.60	0.57	0.68
Resources for emotional/mental wellbeing	0.52			0.70	0.50	0.75
Family respected		0.31		0.79	0.46	0.79
Attention when needed help		0.54		0.53	0.65	0.57
Trust		0.74		0.46	0.64	0.59
Competence		0.62		0.64	0.49	0.76
Washrooms		0.34		0.88	0.28	0.92
Cleanliness		0.41		0.81	0.40	0.84
Safe		0.74		0.55	0.52	0.73
Enough staff		0.31		0.86	0.35	0.88
Wait time for provider			0.63	0.59		0.99
Wait time for labs or drugs			0.65	0.56		0.99
Time with provider			0.25	0.93		1.00
Hold back questions				0.98	0.10	0.99
Pressured				0.98		0.99
Discrimination		0.24		0.96	0.13	0.98
Neglected		0.31		0.89	0.22	0.95
Verbal abuse		0.29	0.26	0.86	0.24	0.94
Bribe				0.99		1.00

Notes: PCANC: Person-centered antenatal care. Blanks represent loading<.1

Supplementary Table 6: Exploratory factor analysis of retained 36 PCANC items with the confirmation sample, Ghana and Kenya, N=1,988

	**3 factor structure for full scale**				**single factor structure for full scale**		**Single factor by sub-scale**	
Subscale/items	*Factor1*	*Factor2*	*Factor3*	*Uniqueness*	*Factor1*	*Uniqueness*	*Factor1*	*Uniqueness*
**Dignity and respect**								
Greeted		0.43		0.63	0.59	0.65	0.59	0.66
Treat you with respect		0.77		0.42	0.66	0.57	0.82	0.33
Friendly care		0.78		0.41	0.66	0.56	0.82	0.33
Knowledge valued		0.50		0.48	0.72	0.49	0.65	0.58
Privacy not exposed	0.31			0.74	0.50	0.75	0.49	0.76
Info confidentiality		0.49		0.73	0.46	0.79	0.51	0.74
Auditory privacy		0.24		0.79	0.44	0.81	0.44	0.81
Verbal abuse		0.27		0.90	0.23	0.95	0.26	0.93
**Communication and Autonomy**								
Introductions by provider	0.41			0.82	0.32	0.90	0.37	0.86
Called preferred name		0.45		0.82	0.35	0.88	0.27	0.93
Heard and listened to		0.52		0.55	0.65	0.58	0.54	0.71
Consent	0.55			0.66	0.54	0.71	0.55	0.70
Involved in decisions	0.54			0.52	0.67	0.55	0.69	0.53
Explain exams/procedures	0.76			0.46	0.63	0.61	0.72	0.48
Explain medications	0.73			0.46	0.66	0.57	0.74	0.46
Told examination results	0.70			0.55	0.54	0.71	0.65	0.58
Told test results	0.76			0.51	0.57	0.68	0.68	0.54
Could ask any questions	0.42			0.56	0.66	0.56	0.66	0.57
Encouraged questions	0.68			0.46	0.68	0.53	0.75	0.44
Questions were answered	0.45			0.55	0.67	0.56	0.68	0.54
Check understood information	0.64			0.46	0.70	0.51	0.74	0.46
Language they understood		0.48		0.78	0.28	0.92	0.17	0.97
Asked about birth plan	0.43			0.76	0.40	0.84	0.42	0.82
**Responsive and Supportive care**								
Wait time for provider			0.62	0.62	0.07	1.00	0.03	1.00
Wait time for labs or drugs			0.67	0.55	-0.09	0.99	-0.13	0.98
Received best care		0.72		0.43	0.69	0.53	0.71	0.49
Physical wellbeing assessed	0.44			0.75	0.47	0.78	0.42	0.82
Mental/emotions wellbeing assessed	0.68			0.56	0.58	0.67	0.56	0.69
Resources for emotional/mental wellbeing	0.59			0.67	0.50	0.75	0.51	0.73
Attention when needed help		0.49		0.54	0.65	0.57	0.67	0.55

Trust	0.73	0.47	0.64	0.59	0.71	0.49
Competence	0.59	0.64	0.49	0.76	0.58	0.66
Washrooms	0.37	0.87	0.28	0.92	0.36	0.87
Cleanliness	0.44	0.79	0.41	0.83	0.48	0.77
Safe	0.76	0.54	0.52	0.73	0.65	0.58
Enough staff	0.31	0.86	0.36	0.87	0.40	0.84

Notes: PCANC: Person-centered antenatal care

Supplementary Table 7: Unstandardized and standardized loadings from the three-factor confirmatory model for the 36-item PCANC scale by country, Ghana and Kenya, N=1,988

Latent variable / observed variable	Ghana (N=992)		Kenya (N=996)	
	Unstandardized (SE)	Standardized (SE)	Unstandardized (SE)	Standardized (SE)
**Dignity and respect**				
Greeted	1	0.67 (0.02)	1	0.57 (0.03)
Treat you with respect	0.81 (0.04)	0.75 (0.02)	0.75 (0.05)	0.63 (0.03)
Friendly care	0.82 (0.04)	0.74 (0.02)	0.84 (0.06)	0.66 (0.03)
Knowledge valued	1.14 (0.06)	0.79 (0.02)	1.02 (0.07)	0.68 (0.03)
Privacy not exposed	0.73 (0.05)	0.54 (0.03)	1.04 (0.09)	0.51 (0.03)
Info confidentiality	0.67 (0.05)	0.51 (0.03)	0.53 (0.05)	0.43 (0.03)
Auditory privacy	0.68 (0.06)	0.39 (0.03)	1.08 (0.08)	0.52 (0.03)
Verbal abuse	0.21 (0.03)	0.28 (0.03)	0.11 (0.02)	0.17 (0.03)
**Communication and autonomy**				
Introductions by provider	1	0.28 (0.03)	1	0.35 (0.03)
Called preferred name	1.36 (0.19)	0.40 (0.03)	1.05 (0.14)	0.35 (0.03)
Heard and listened to	2.12 (0.25)	0.67 (0.02)	1.55 (0.16)	0.62 (0.03)
Consent	1.16 (0.25)	0.55 (0.02)	2.07 (0.21)	0.55 (0.03)
Involved in decisions	2.42 (0.28)	0.79 (0.01)	1.86 (0.20)	0.56 (0.03)
Explain exams/procedures	2.33 (0.28)	0.72 (0.02)	2.29 (0.24)	0.63 (0.03)
Explain medications	2.40 (0.28)	0.75 (0.02)	2.13 (0.22)	0.63 (0.02)
Told examination results	1.99 (0.24)	0.60 (0.02)	1.87 (0.21)	0.50 (0.03)
Told test results	2.12 (0.25)	0.64 (0.02)	1.86 (0.21)	0.51 (0.03)
Could ask any questions	2.06 (0.25)	0.66 (0.02)	2.25 (0.23)	0.71 (0.02)
Encouraged questions	2.56 (0.30)	0.74 (0.02)	2.50 (0.25)	0.68 (0.02)
Questions were answered	2.16 (0.26)	0.68 (0.02)	1.78 (0.19)	0.68 (0.02)
Check understood information	2.65 (0.31)	0.82 (0.01)	2.20 (0.23)	0.62 (0.02)
Language they understood	0.83 (0.11)	0.43 (0.03)	0.09 (0.04)	0.08 (0.04)
Asked about birth plan	1.56 (0.19)	0.40 (0.03)	1.47 (0.18)	0.41 (0.03)
**Responsive and supportive care**				
Received best care	1	0.84 (0.01)	1	0.69 (0.02)
Wait time for provider	-0.07 (0.04)	-0.05 (0.03)	0.33 (0.06)	0.21 (0.03)
Wait time for labs or drugs	-0.38 (0.06)	-0.21 (0.03)	0.22 (0.06)	0.12 (0.03)
Physical wellbeing assessed	0.70 (0.05)	0.49 (0.03)	0.94 (0.08)	0.41 (0.03)
Mental/emotions wellbeing assessed	1.19 (0.07)	0.56 (0.03)	1.15 (0.10)	0.48 (0.03)
Resources for emotional/mental wellbeing	1.14 (0.07)	054 (0.03)	0.75 (0.09)	0.33 (0.04)
Attention when needed help	0.98 (0.04)	0.77 (0.02)	0.95 (0.06)	0.60 (0.03)
Trust	1.06 (0.04)	0.81 (0.01)	0.97 (0.05)	0.63 (0.02)
Competence	0.80 (0.03)	0.67 (0.02)	0.66 (0.05)	0.50 (0.03)
Washroom availability	0.82 (0.06)	0.46 (0.03)	0.31 (0.07)	0.16 (0.04)
Cleanliness	0.76 (0.05)	0.49 (0.03)	0.81 (0.08)	0.40 (0.03)
Safe	0.89 (0.04)	0.65 (0.02)	0.85 (0.05)	0.64 (0.02)
Enough staff	0.50 (0.05)	0.33 (0.03)	0.95 (0.08)	0.44 (0.03)

Notes: PCANC: Person-centered antenatal care. SE: Standard error

Supplementary Table 8: Goodness-of-fit indicators for different three-factor confirmatory models for the PCANC scale, Ghana and Kenya, N=1,988

Good-of Fit Statistic	Different models considering the levels of modification indices included					
	36-items			20-items		
	Model 1	Model 2	Model 3	Model 4	Model 5	Model 6
Chi-square, p-value	11822.102, p<0.001	3523.978, p<0.001	2150.827, p<0.001	4917.535, p<0.001	1524.876, p<0.001	694.801, p<0.001
Root mean squared error of approximation	0.098	0.055	0.048	0.118	0.070	0.054
P of close fit	<0.001	<0.001	0.922	<0.001	<0.001	0.050
Comparative fit index	0.627	0.900	0.941	0.700	0.913	0.963
Tucker–Lewis index	0.604	0.876	0.903	0.665	0.883	0.931
Standardized root mean squared residual	0.220	0.057	0.046	0.242	0.062	0.041

Notes: PCANC: Person-centered antenatal care
Model 1 - *Model with 36 observed variables without modification indices*
Model 2- *Model with 36 observed variables with modification indices whose covariates is 30 or more*
Model 3 - *Model with 36 observed variables with modification indices whose covariates is 10 or more*
Model 4 - *Model with 20 observed variables without modification indices*
Model 5- *Model with 20 observed variables with modification indices whose covariates is 30 or more*
Model 6 - *Model with 20 observed variables with modification indices whose covariates is 10 or more*
Note – *Model with 36 observed variables with modification indices whose covariates is 5 or more failed to run with an error of too many options, however Good of fit statistics suggest that an excellent model is the one with the modification indices, as observed increasing or lowering the covariance cutoff for inclusion improves model fit*

Supplementary Table 9: Characteristics of the PCANC scale and sub-scales in the confirmation sample, Ghana and Kenya, N=1,988

	Internal consistency reliability			*Standardized scores*			
	Number of items	***Average interitem correlation***	***Scale reliability coefficient***	***Mean***	***Std. dev.***	***Min***	***Max***
Combined (N=1988)							
Full 36-item scale	36	0.25	0.92	71.50	16.35	12.04	100
34-item scale*	34	0.28	0.93	71.13	17.26	10.78	100
20-item short scale	20	0.27	0.89	70.60	18.36	1.67	100
Dignity and respect subscale	8	0.21	0.78	81.23	17.22	8.33	100
Communication and autonomy subscale	15	0.37	0.88	65.23	21.54	4.44	100
Responsive and supportive care subscale	13	0.22	0.78	72.75	15.16	15.38	100
Responsive and supportive care subscale*	11	0.30	0.82	71.81	17.55	0.00	100
Kenya (N=996)							
Full 36-item scale	36	0.22	0.91	68.84	15.78	21.30	100
34-item scale*	34	0.24	0.91	68.15	16.43	20.59	100
20-item short scale	20	0.27	0.88	67.27	17.53	15.00	100
Dignity and respect subscale	8	0.29	0.77	79.69	17.33	20.83	100
Communication and autonomy subscale	15	0.28	0.86	60.36	21.00	13.33	100
Responsive and supportive care subscale	13	0.19	0.75	71.94	14.40	17.95	100
Responsive and supportive care subscale*	11	0.23	0.77	70.38	15.88	18.18	100
Ghana (N=992)							
Full 36-item scale	36	0.30	0.94	74.18	16.47	12.04	100
34-item scale*	34	0.33	0.94	74.11	17.56	10.78	100
20-item short scale	20	0.37	0.92	73.95	18.59	1.67	100
Dignity and respect subscale	8	0.37	0.82	82.77	16.99	8.33	100
Communication and autonomy subscale	15	0.38	0.90	70.12	20.97	4.44	100
Responsive and supportive care subscale	13	0.30	0.85	73.56	15.85	15.38	100
Responsive and supportive care subscale*	11	0.37	0.87	73.26	18.98	0.00	100

Notes: PCANC: Person-centered antenatal care. The 34-item full scale and 11-item responsive and supportive care sub-scale exclude the two wait time variables

Supplementary Table 10: All PCANC questions, with final decisions and reasons for exclusion

No	Label	Question	Decision for 36 item scale	Reason for inclusion/exclusion from 36 item scale	Decision for 20-item short scale
1	Greeted	Did they greet you well when you arrived or responded well if you greeted first?	Included	Adequate performance	Included
2	Treat you with respect	Did they treat you with respect?	Included	Adequate performance	Included
3	Friendly care	Were they friendly or nice to you?	Included	Adequate performance	Included
4	Knowledge valued	Did you feel your knowledge was valued? Did they appreciate or accept your ideas or knowledge about your health?	Included	Adequate performance	Excluded
5	Privacy not exposed	During physical exams (like abdominal and pelvic exams) were you covered up with a cloth or blanket or screened with a curtain so that you did not feel exposed?	Included	Adequate performance	Included
6	Info confidentiality	Did you feel your health information was kept confidential by the health workers?	Included	Adequate performance	Included
7	Auditory privacy	Did you feel you could discuss your problems with the health workers, without others not involved in your care overhearing your conversations?	Included	Adequate performance	Excluded
8	Family respected	Did the health workers respect your family or companions who were with you?	Excluded	Excluded because of high not applicable	Excluded
9	Verbal abuse	Did you feel they talked to you badly (For example, shouted at you, scolded, insulted, or threatened, you)?	Included	Moderate performance but retained for conceptual relevance	Excluded
10	Discrimination	Did you feel discriminated against by the health workers in any way? I.e., did you feel you were treated differently based on something about you?	Excluded	Excluded because of low loading	Excluded
11	Neglected	Did you feel the health workers avoided, ignored, or neglected you?	Excluded	Excluded because of low loading	Excluded
12	Introductions by provider	Did the health workers introduce themselves to you when they first saw you?	Included	Adequate performance	Included
13	Called preferred name	Did they call you by your name (or appropriately)?	Included	Adequate performance	Included
14	Heard and listened to	Did you feel heard by the health workers?	Included	Adequate performance	Included
15	Consent	Did the health workers ask your permission before examining or doing procedures on you?	Included	Moderate performance	Included
16	Involved in decisions	Did the health workers involve you in decisions about your care?	Included	Adequate performance	Included
17	Explain exams/procedures	Did they explain to you why they were doing examinations or tests on you?	Included	Adequate performance	Included
18	Understood tests	Did you understand the purpose of any examinations or tests you were asked to do? ... like urine or	Excluded	Excluded because of correlation with explain exams/procedures	Excluded

		blood tests, ultrasound, etc., that you were asked to do including those you were asked to do outside the facility)?			
19	Told examination results	Did they tell you the results of examinations you did like your blood pressure and weight?	Included	Adequate performance	Excluded
20	Told test results	Did they tell you the results of tests you did like blood or urine tests?	Included	Adequate performance	Excluded
21	Explain medications	Did they explain to you why they were giving or prescribing you any medicine?	Included	Adequate performance	Included
22	Understood medicines	Did you understand the purpose of any medicines you were given or prescribed to go and buy?	Excluded	Excluded because of correlation with explain medicines	Excluded
23	Could ask any questions	Did you feel you could ask the health workers any questions you had?	Included	Adequate performance	Excluded
24	Encouraged questions	Did they encourage you to ask questions?	Included	Adequate performance	Included
25	Hold back questions	Did you hold back from asking questions for any reason?	Excluded	Excluded because of low loading	Excluded
26	Questions were answered	Do you feel your questions were answered when you asked them?	Included	Adequate performance	Excluded
27	Check understood information	Did they check that you understood information that was given to you?	Included	Adequate performance	Included
28	language they understood	Did the health workers speak to you in a language you could understand or using words you could understand?	Included	Adequate performance	Excluded
29	Asked about birth plan	Did a health worker discuss your birth plan with you (e.g., where you wanted to give birth or what you wanted during the birth?)	Included	Adequate performance	Excluded
30	Pressured	Did you feel forced into a decision by health workers?	Excluded	Excluded because of low loading	Excluded
31	Wait time for provider	How did you feel about the amount of time you had to wait to be seen by a health worker during antenatal visits (i.e., the time from when you arrived at the health facility to when you saw the midwife or doctor)?	Included	Included despite low loading because of conceptual relevance	Excluded
32	Wait time for labs or drugs	How did you feel about the amount of time it took you to get your labs done and/or get your drugs at the facility?	Included	Included despite low loading because of conceptual relevance	Excluded
33	Time with provider	How did you feel about the amount of time the doctor or midwife spent with you? (i.e., was it rushed or did they take their time with you)	Excluded	Excluded because of low loading	Excluded
34	Received best care	Did you feel they took the best care of you?	Included	Adequate performance	Included
35	Showed they cared	Did you feel the health workers cared about you?	Excluded	Excluded because of correlation with best care	Excluded

36	Physical wellbeing assessed	Did they ask you about your physical health?	Included	Adequate performance	Excluded
37	Mental/emotional wellbeing assessed	Did they ask you about your mental and emotional health?	Included	Adequate performance	Included
38	Resources for emotional/mental wellbeing	Did they give you the support to deal with your mental and emotional health?	Included	Adequate performance	Excluded
39	Attention when needed help	When you needed support from the providers with regards to your health, did they pay attention?	Included	Adequate performance	Excluded
40	Trust	Did you feel you could trust the health workers with regards to your care?	Included	Adequate performance	Included
41	Competence	Did you feel the health workers were competent?	Included	Adequate performance	Excluded
42	Washrooms	Could you use the washrooms in the facility if you needed to?	Included	Adequate performance	Included
43	Cleanliness	Did you feel the health facility environment, including the washrooms were clean?	Included	Adequate performance	Included
44	Safe	In general, did you feel safe (physically and psychologically) in the place you received antenatal care?	Included	Adequate performance	Included
45	Enough staff	Do you think there were enough health staff in the facility to care for you?	Included	Adequate performance	Excluded
46	Bribe	During your antenatal care, did any health worker at the facility ask you or your family for an unofficial payment?	Excluded	Excluded because of low loading	Excluded
47	Physical abuse	Did you feel they handled you badly (For example pushed, beat, slapped, pinched or physically restrained you)?	Excluded	Excluded because of low frequency and loading	Excluded
48	Language level they understood	Did they speak to you using words you could understand?	Excluded	Combined with language they understood	Excluded
49	Preferred clinic	Were you able to go to your preferred place/clinic for antenatal care?	Excluded	Excluded because of low relevance	Excluded
50	Preferred provider	Were you able to see your preferred provider for antenatal care?	Excluded	Excluded because of low relevance	Excluded

Notes:

1. *PCANC: Person-centered antenatal care*
2. *Last four questions not included in the CPIPE trial.*
3. *Response options for all question are: 0, No, never* | *1, Yes, a few times* | *2, Yes, most of the time* | *3, Yes, all the time, except those noted below*
4. *Questions 9, 10, 11, 47 response options are: 0, No, never* | *1, Yes, once* | *2, Yes, a few times* | *3, Yes, many times*
5. *Questions 1, 12, 28, 41 response options are: 0, No, none of them* | *1, Yes, a few of them* | *2, Yes, most of them* | *3, Yes, all of them*
6. *Questions 31, 32 response options are: 0, It was just right* | *1, It was somewhat long* | *2, It was very long* | *3, It was extremely long*
7. *Question 33 response options are: 0, It was just right* | *1, It was too long* | *2, It was somewhat short* | *3, It was very short*
8. *Some questions also include option 4, not applicable/don't know, which need to be recoded before scoring (see supplementary table 4)*
